# Human Immunodeficiency Virus 1 Preferentially Fuses with pH-Neutral Endocytic Vesicles in Cell Lines and Human Primary CD4+ T-Cells

**DOI:** 10.1021/acsnano.3c05508

**Published:** 2023-08-17

**Authors:** Manish Sharma, Mariana Marin, Hui Wu, David Prikryl, Gregory B. Melikyan

**Affiliations:** † Department of Pediatrics, Division of Infectious Diseases, Emory University School of Medicine, Atlanta, Georgia 30322, United States; ‡ Children’s Healthcare of Atlanta, Atlanta, Georgia 30322, United States

**Keywords:** single virus tracking, virus labeling, membrane fusion, endocytosis, pH-sensing

## Abstract

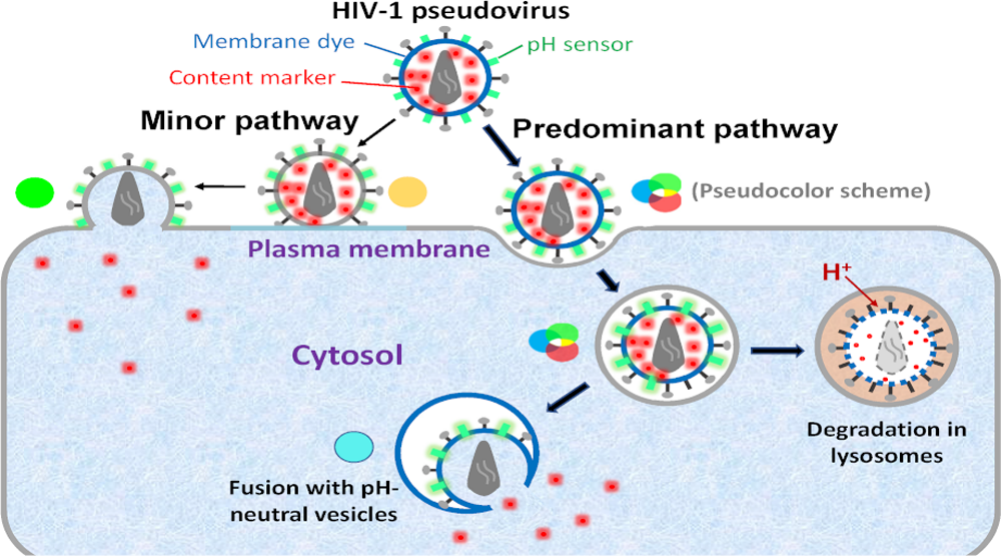

Despite extensive efforts, the principal sites of productive HIV-1 entry in different target cells—plasma membrane (PM) vs endosomes—remain controversial. To delineate the site(s) of HIV-1 fusion, we implemented a triple labeling approach that involves tagging pseudoviruses with the fluid-phase viral content marker, iCherry, the viral membrane marker, DiD, and the extraviral pH sensor, ecliptic pHluorin. The viral content marker iCherry is released into the cytoplasm upon virus–cell fusion irrespective of the sites of fusion. In contrast, the extent of dilution of the membrane marker upon fusion with the PM (loss of signal) vs the endosomal membrane (no change in punctate DiD appearance) discriminates between the principal sites of viral fusion. Additionally, ecliptic pHluorin incorporated into the viral membrane reports whether virus fusion occurs in acidic endosomes. Real-time single virus imaging in living HeLa-derived cells, a CD4+ T-cell line, and activated primary human CD4+ T-cells revealed a strong (80–90%) HIV-1 preference for fusion with endosomes. Intriguingly, we observed HIV-1 fusion only with pH-neutral intracellular vesicles and never with acidified endosomes. These endocytic fusion events are likely culminating in productive infection since endocytic inhibitors, such as EIPA, Pitstop2, and Dynasore, as well as a dominant-negative dynamin-2 mutant, inhibited HIV-1 infection in HeLa-derived and primary CD4+ T-cells. Furthermore, the inhibition of endocytosis in HeLa-derived cells promoted hemifusion at the PM but abrogated complete fusion. Collectively, these data reveal that the primary HIV-1 entry pathway in diverse cell types is through fusion with pH-neutral intracellular vesicles.

HIV-1 fusion with the host cell membrane releases the viral nucleocapsid into the cytoplasm, thereby initiating a cascade of downstream events that establish a productive infection. HIV-1 fusion is initiated upon sequential interactions of the envelope glycoprotein (Env) with the CD4 receptor and coreceptors, CCR5 or CXCR4.^1^ The formation of a ternary Env-CD4-coreceptor complex triggers the refolding of the transmembrane gp41 subunit into the six-helix bundle (6HB) structure that promotes the fusion pore formation.^2^ While the mechanism of HIV-1 Env-mediated fusion is reasonably well understood, the virus’ preferred entry pathway(s) into cells remain controversial, with evidence supporting fusion with both the PM and endosomes in different cell types.^2−4^

The original argument for direct HIV-1 fusion with the PM is based on the lack of low pH-dependence for Env-mediated membrane fusion, so the internalized virions were thought to be degraded by cells.^5−8^ This notion is supported by efficient Env-mediated syncytia formation^9^ and virus-mediated fusion between two adjacent cells, referred to as fusion-from-without.^10^ However, the ability to fuse at neutral pH does not preclude a virus from entering and fusion with acidified endosomes. Additional arguments include: (a) coreceptor-independence of bulk HIV-1 uptake by cells, irrespective of productive entry,^11^ (b) lack of correlation between ligand-mediated CD4 or coreceptor endocytosis and HIV-1 infection,^7,12^ (c) the ability to infect cells that do not support efficient endocytosis or under conditions inhibiting endocytosis,^13^ (d) lack of colocalization of intracellular HIV-1 particles with both CD4 and coreceptors,^11^ and (e) block of HIV-1 fusion/infection by gp41-derived inhibitory peptide added after prolonged incubation of viruses and cell at reduced temperature permissive for virus uptake but not fusion.^11^

On the other hand, several lines of evidence support HIV entry via an endocytic pathway. Inhibition of clathrin and dynamin-2-mediated endocytosis in HeLa-derived cells reduces the efficacy of HIV-cell fusion and infection.^13−16^ In addition, raising endosomal and lysosomal pH tends to enhance HIV-1 infectivity, apparently by sparing the virus from degradation in lysosomes^17,18^ Experimental evidence supports productive HIV-1 entry via endocytosis in other cell types, such as CD4+ T cells, astrocytes, and macrophages.^19,20^ Additional evidence came from a screen for HIV-1 host dependence factors involved in endocytic trafficking that we carried out. This screen identified several proteins, including Rab5A, Snx3 and Snx10, knockdown of which in a CD4+ T-cell line significantly reduced HIV-1 fusion and infection.^21^ Intriguingly, HIV-1 fusion in endosomes has been shown in target cells after virological synapse mediated transfer from donor cells.^22^ The common caveats of arguments for and against endocytic HIV-1 entry are the reliance on bulk virus uptake (and fusion) and assessment of the virus’ apparent colocalization with intracellular markers without following the functionally relevant infectious events. An important limitation is the lack of direct approaches to reliably define the sites of HIV-1 fusion. As a result, the HIV-1 entry pathways into different cell types remain unresolved.

We have previously introduced an experimental approach to determine the sites of HIV-1 fusion by colabeling pseudoviruses with the fluorescent lipophilic dye, DiD (incorporated into the viral lipid bilayer), and a GFP-based viral content marker that is released from particles into the cytoplasm following the virus fusion.^15^ Using time-resolved imaging of single virus fusion with live cells, we tracked viral particles undergoing fusion, which is manifested by the loss of viral content regardless of whether fusion occurs at the cell surface or in endosomes. By contrast, fusion with the PM leads to the disappearance of DiD fluorescence due to its marked dilution, whereas fusion with the endosomal membrane results in limited dilution of a lipid dye which retains its punctate appearance.^15^ Using this approach, we have found that pseudoviruses consistently fused with endosomes in HeLa-derived and CD4+ T-cells, as evidenced by the loss of viral content and retention of the membrane marker.^15^

Here, we implemented a triple labeling approach to pinpoint the sites of HIV-1 fusion in cells. Similar to our previously published approach, pseudoviruses were labeled with a fluid-phase viral content marker that is released upon fusion^15,23^ and a viral lipid marker to discriminate between fusion with the PM vs endosomes.^15^ In addition, we anchored a pH-sensitive green fluorescent protein to the viral membrane to track virus entry into acidic endosomes.^24,25^ This virus labeling approach enabled pinpointing the sites of HIV-1 fusion and revealed a striking preference for fusion with pH-neutral endocytic compartments in epithelial cells, CD4+ T-cell line, and primary human CD4+ T-cells.

## Results

### A Triple Labeling Approach to Pinpoint the Sites of HIV-1 Pseudoparticle Fusion with Cells

**Figure 1:**
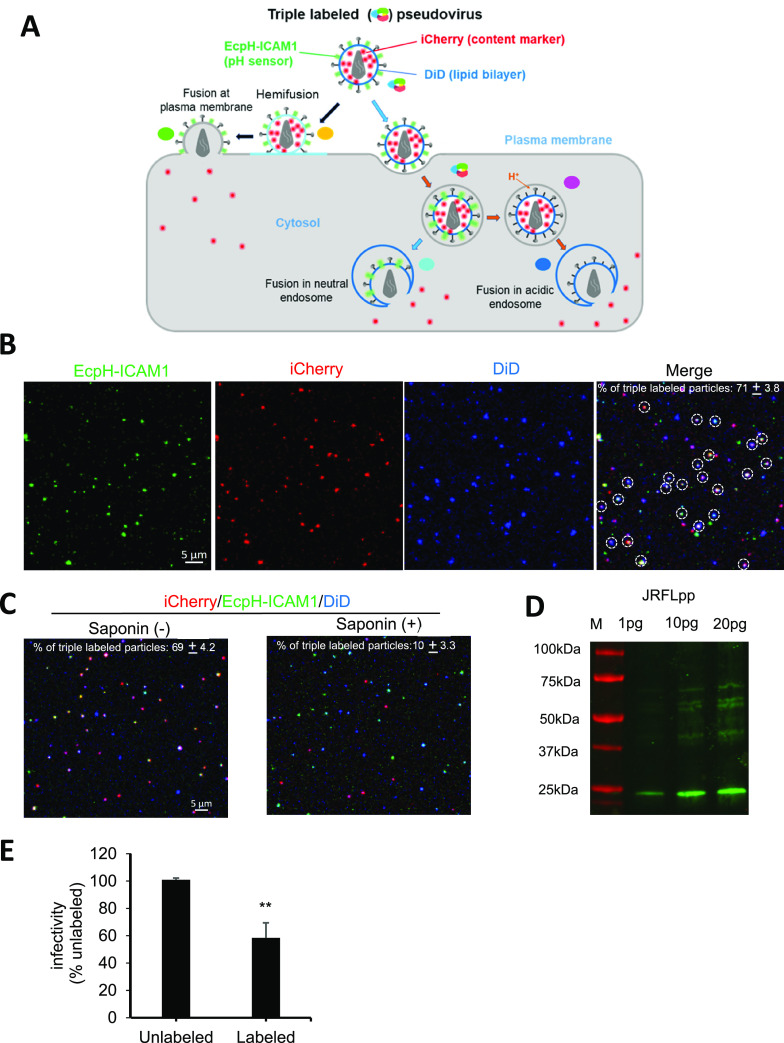
HIV-1 labeling with three fluorescent markers for defining the sites of viral fusion in cells. (A) Illustration of the strategy to discriminate between possible HIV-1 fusion sites in cells. In brief, HIV-1 pseudoviruses bearing the HIV-1 envelope glycoprotein are labeled with the viral lipid marker, DiD (blue), the virus surface-exposed pH sensitive EcpH-ICAM1 probe (green) and the fluid phase marker for viral content, iCherry (red). Using this labeling scheme, three distinct outcomes can be envisioned. Fusion with the PM is manifested by a loss of DiD and iCherry signals due to their dilution in the PM and the cytoplasm, respectively, while the EcpH signal should remain unchanged due to a constant neutral extracellular pH. Fusion with the PM thus results in the transition from white to green color. By contrast, fusion with the endosomes should lead to a loss of iCherry but not DiD signal, since the latter redistributes into the endosomal membrane and thus remains localized. Whether the EcpH signal is lost depends on the endosomal pH: it should remain unchanged upon fusion with pH-neutral compartments (transition from white to cyan color) or quenched, if fusion occurs in acidified endosomes (white-to-blue transition). (B) The efficiency of triple labeling of HIV-1pp was assessed by confocal imaging of pseudoviruses attached to coverslips. White circles mark triple labeled particles. The average percent of colocalization of all three markers is shown on the merged image panel (calculated for 3 image fields each containing ∼300 particles). (C) Coverslip attached single virus particles before and after lysis by saponin (100 μg/mL). (D) p24 Western blot shows Gag processing upon maturation of control and triple labeled HIV-1 (JRFL) pseudoviruses. (E) Infectivity of unlabeled and labeled (iCherry, EcpH-ICAM1 and DiD) pseudoviruses in TZM-bl cells. Data are mean and SD of two different viral stocks, and each experiment was performed in triplicate. Data were analyzed by Student’s *t* test. **, *p* < 0.01.

To delineate the HIV-1 fusion site in living cells, we designed a triple labeling approach to incorporate three different fluorescent tags into HIV-1 pseudoviruses that include iCherry (viral content marker), ecliptic pHluorin-ICAM-1 (ecliptic pHluorin (EcpH) fused to N-terminus of the human ICAM-1 transmembrane domain), and DiD (viral membrane marker) (Figure 1A). Free iCherry produced from Gag-iCherry upon HIV-1 maturation acts as the virus content marker and is released into the cytoplasm upon fusion.^23^ Importantly, the extent of dilution of the lipophilic dye, DiD, upon fusion discriminates between virus fusion with the PM (nearly infinite dilution and loss of signal) vs endosomes (limited dilution and retention of punctate signal).^15^ Although HIV-1 fusion does not require low pH to mediate membrane fusion,^5,6^ this does not preclude a virus from fusing with acidified endosomes. We therefore incorporated EcpH-ICAM1, which is quenched at low pH, into pseudoviruses to report their delivery into acidified endosomes.^25^

Using this approach, we generated and characterized triple labeled HIV-1 JRFL or HXB2 Env pseudotyped particles (JRFLpp and HXB2pp). Visualization of the coverslip-immobilized viruses revealed that ∼70% JRFLpp or HXB2pp was triple labeled with iCherry, EcpH-ICAM1 and DiD (Figures 1B and S1A, white circles). We assessed the extent of Gag-iCherry cleavage in triple labeled particles by permeabilizing their membranes with saponin. Around 85% of triple labeled particles contained cleaved/releasable iCherry, as evidenced by a loss of iCherry upon the addition of saponin (Figures 1C and S1B). Triple labeling did not affect the HIV-1 particle maturation (Figures 1D and S1C). We also found that coincorporation of iCherry/EcpH-ICAM1 and DiD modestly reduces specific infectivity compared to unlabeled particles (Figures 1E and S1D).

### HIV-1 Pseudoviruses Preferentially Fuse with pH- Neutral Endocytic Vesicles in TZM-bl Cells

**Figure 2:**
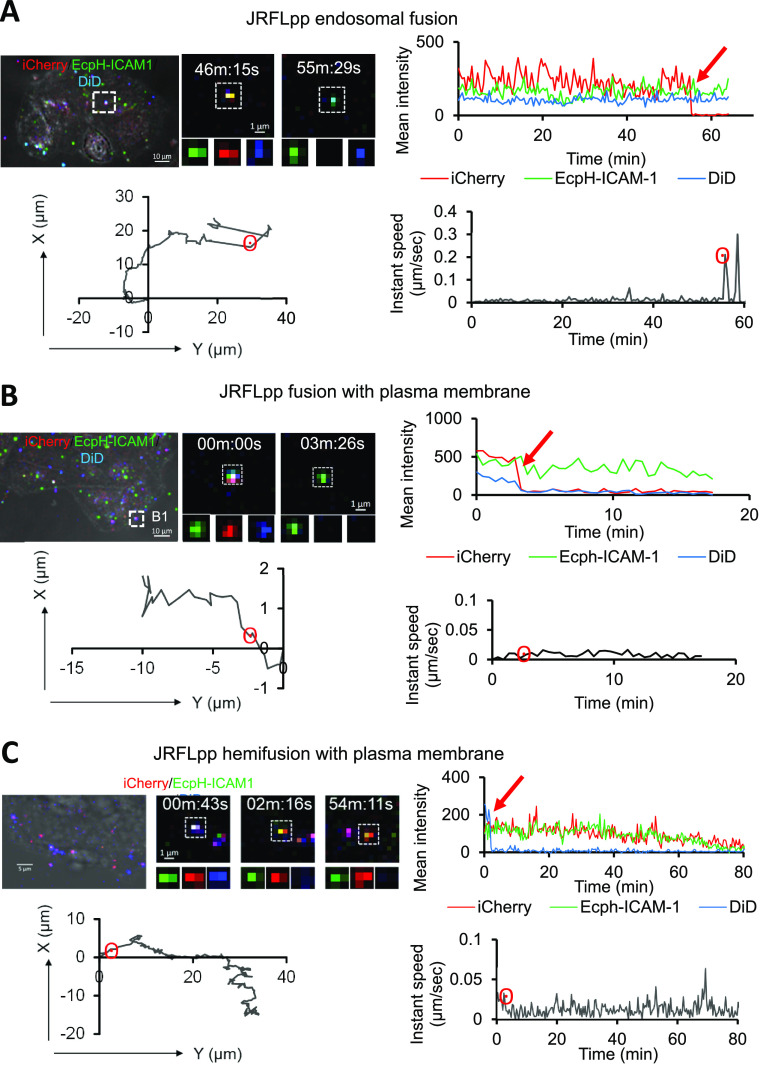
Fusion sites of HIV-1 JRFL Env pseudoviruses in TZM-bl cells. (A, B) Examples of JRFLpp fusion in pH-neutral endocytic versicles and with PM of TZM-bl cells, respectively. Cells were allowed to bind triple labeled pseudoviruses in the cold, and virus-cell fusion was synchronously initiated by quickly shifting to 37 °C and imaged by time-lapse confocal microscopy (top left). Fluorescence intensity profiles (top right), X–Y trajectories (bottom left), and instant velocities (bottom right) of single JRFLpp obtained by single-particle tracking. Single JRFLpp fusion is detected by a loss of iCherry at 55:30 min (A) and ∼3:26 min (B), without or with a concomitant loss of DiD signal, respectively (see Movies S1 and S2). (C) A hemifusion event (loss of DiD without a loss of iCherry). Fluorescence profiles, the X–Y trajectory, and instant velocity are shown (see Movie S3). The points of fusion/hemifusion are marked on X–Y trajectories and instant velocity plots by red circles (see Movie S3). Red arrows on fluorescence intensity plots mark virus fusion and hemifusion events.

**Figure 3:**
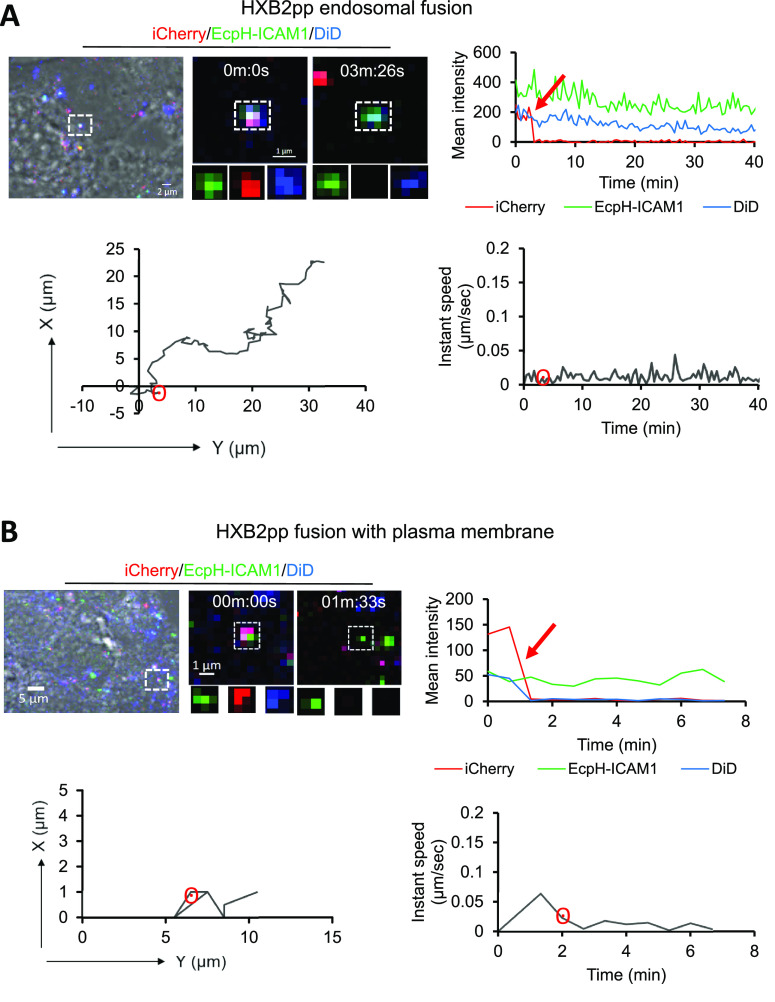
Fusion sites of HIV-1 HXB2 Env pseudoviruses in TZM-bl cells. (A, B) Examples of triple labeled HXB2pp fusion in neutral endocytic vesicles and PM (top left). Fluorescence traces (top right), X–Y trajectories (bottom left), and instant velocities (bottom right) of single HXB2pp fusion (loss of iCherry at 3:26 min (A) and 1:3 min (B)) are shown (see Movies S4 and S5). DiD signal is lost in B but not in A, consistent with endosomal and PM fusion events, respectively. Red arrows on fluorescence intensity plots mark virus fusion and hemifusion events.

We first imaged single HIV-1 fusion with the HeLa-derived indicator TZM-bl cells expressing high levels of CD4 and CCR5 and an endogenous level of CXCR4. Triple labeled JRFLpp or HXB2pp were prebound to cells in the cold, and virus entry/fusion was synchronously initiated by shifting to 37 °C. Live-cell imaging of triple labeled JRFLpp revealed three distinct types of fusion events (Figure 2). First, we typically observed JRFLpp fusion with the endosome manifested by a loss of iCherry, but no changes in DiD or EcpH-ICAM-1 fluorescence were detected at that point (Figure 2A, Movie S1). The steady EcpH-ICAM-1 signal implies that this fusion event occurred in a pH-neutral compartment. A surge in the particle’s velocity at the time of fusion (Figure 2A, bottom right panel, marked by a red circle) further supports the notion that viral particle was internalized and trafficked in an endosome. Second, fusion with the PM was detected as a synchronous disappearance of iCherry and DiD signals without loss of EcpH signal (Figure 2B, Movie S2). This virus exhibited limited movement expected for a particle attached to the cell surface (Figure 2B, bottom right panel). Third, we observed rare virus hemifusion events:^15^ loss of DiD fluorescence (lipid mixing with PM), without a loss of iCherry or EcpH, and without significant pseudovirus movement at the time of hemifusion (Figure 2C, Movie S3). Similar to the CCR5-tropic JRFL Env, the CXCR4 tropic HIV-1 HXB2 Env also mediated viral fusion at two distinct sites in TZM-bl cells–endosomes and the PM (Figure 3A, B, Movies S4 and S5).

**Figure 4:**
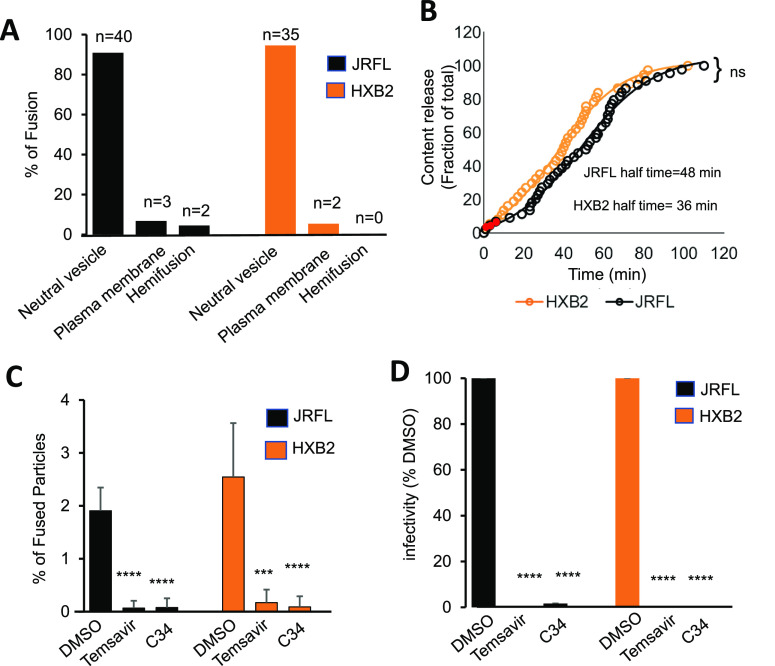
Neutral endocytic vesicles are the prevalent HIV-1 fusion sites in TZM-bl cells. (A) The distribution of HIV-1 fusion sites (N is the number of fusion events in each category). (B) The kinetics of single HIV-1 fusion. Red circles mark the PM fusion events. Half-time of fusion was determined by sigmoidal curve fitting. (C) The fraction of single HIV-1 fusion (out of total cell-bound particles) and (D) HIV-1 infectivity was measured across multiple experiments. Temsavir (10 μM) and C34 peptide (400 nM), potent HIV-1 entry inhibitors, were present in control experiments. Data are mean ± SD from 5 to 15 experiments. Data were analyzed by Student’s *t* test. ***, *p* < 0.001, ****, *p* < 0.0001.

Analysis of single HIV-1 fusion events in TZM-bl cells revealed that, strikingly, ∼90% of triple labeled HIV-1 particles (pseudotyped with either JRFL or HXB2) fused with pH-neutral endocytic vesicles, while only ∼6% of particles fused with PM (Figure 4A). Abrupt loss of the iCherry signal upon single virus fusion after initiating synchronized virus entry into TZM-bl cells allowed the measurement of the fusion kinetics. Interestingly, the few HIV-1 pseudovirus fusion events at the PM occurred relatively quickly, within 8–10 min of initiating infection, while the half-time for fusion with endosomes was ∼40 min (Figure 4B). This marked difference is consistent with kinetic competition between fusion with the PM and endocytosis, so that only the faster fusion events can take place at the cell surface before the virus is internalized. We also assessed the fusion efficiency by quantifying percent of fused HIV-1 particles. The extent of JRFL and HXB2 driven fusion was relatively low (∼2–3%) and comparable with the previous reports^26^ (Figure 4C). HIV-1 fusion and infectivity were blocked in the presence of Temsavir, a potent HIV-1 fusion inhibitor that blocks CD4-Env interaction,^27^ and C34 peptide, which is derived from the C-terminal gp41 heptad repeat and blocks the gp41 refolding into the final six-helix bundle structure^28^ (Figure 4C,D). These results demonstrate that loss of iCherry occurred upon HIV-1 Env mediated fusion with cell.

In contrast to the predominant HIV-1 fusion with pH-neutral vesicles, we did not observe any fusion in the acidic compartments. To determine whether the lack of detectable HIV-1 fusion with acidified endosomes was because virus access to these compartments was restricted, we measured the change in extraviral pH using EcpH quenching as a reporter. Overall, ∼30% of all TZM-bl cells-associated particles entered acidic endosomes within 90 min of infection (Figure S2A). We followed a subset of 65 single JRFLpp and found that none of these pseudoviruses fused with acidified endosomes. Two representative JRFLpp shown in Figure S2B (labeled B’ and B’’) exhibited EcpH quenching without loss of DiD or iCherry, demonstrating entry into acidified endosomes without fusion (see also Movie S6). Thus, HIV-1 has access to but fails to fuse with acidified endosomes.

**Figure 5:**
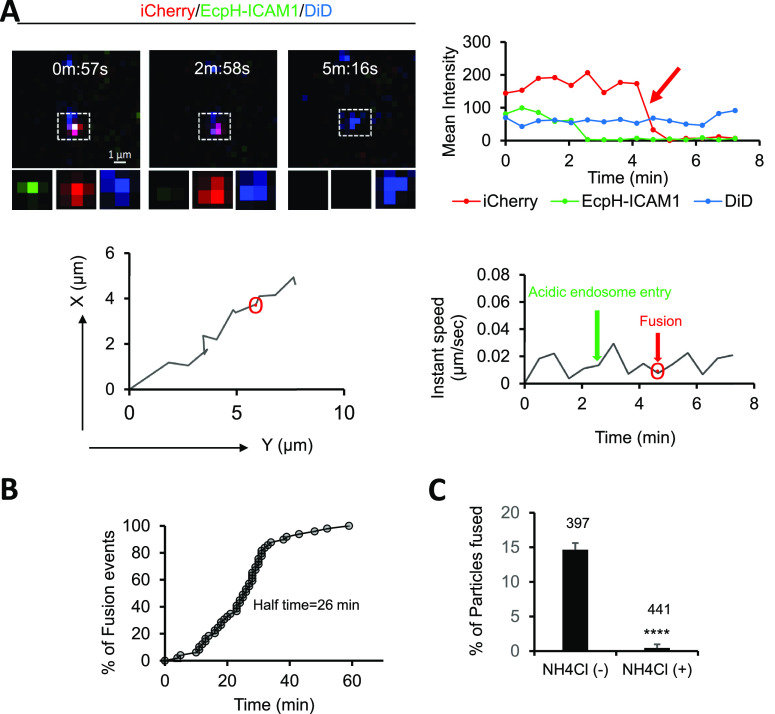
Single VSV-G pseudovirus fusion in acidified endosomes. TZM-bl cells were infected with triple labeled VSV-G pseudoviruses, and single virus fusion (release of iCherry) was detected by single particle tracking in live cells. (A) Time-lapse images (top left), fluorescence traces (top right, red arrow marks virus fusion), X–Y trajectory (bottom left) and instant velocity (bottom right) of a single VSV-G fusion event are shown. EcpH (pH-sensor) quenching occurs at 2:58 min, followed by iCherry release at 4:65 min (see Movie S6). These events, along with the retention of DiD signal, demonstrate that fusion occurred in the acidified endosome. (B) The kinetics of single VSV-G pseudovirus fusion in TZM-bl cells. (C) Single VSV-G pseudovirus fusion in the absence and presence of NH_4_Cl, which raises the endosomal pH. The efficiency of viral fusion in untreated (*n* = 58 out of 397) and ammonium chloride treated (*n* = 2 out of 441) cells is plotted as percent of total cell-associated triple labeled particles. Data from 4 independent experiments were analyzed by Student’s *t* test. ****, *p* < 0.0001.

To verify that our triple labeling strategy can detect virus fusion with acidified endosomes, we pseudotyped HIV-1 particles with VSV-G glycoprotein which mediates membrane fusion at low pH.^29,30^ VSV-G pseudoparticles (VSVpp) were effectively colabeled with iCherry, EcpH-ICAM1 and DiD (Figure S3A), and this labeling did not affect particle maturation or iCherry cleavage (Figure S3B, C). The infectivity was only modestly reduced by this labeling protocol (Figure S3D). As expected, single VSVpp tracking in TZM-bl cells,^31^ revealed that quenching of EcpH-ICAM-1 fluorescence by low pH preceded the viral fusion detected as a loss of iCherry (Figure 5A, Movie S7). Also, consistent with virus-endosome fusion, DiD fluorescence was not lost because the lipid dye was redistributed to the endosomal membrane and remained localized. The half-time of single VSVpp fusion (∼26 min, Figure 5B) was comparable with previous studies.^26^ Around 15% of triple labeled VSVpp fused with these cells during a 1 h imaging session, and viral fusion was abrogated in the presence of ammonium chloride (NH_4_Cl), which raises the endosomal pH (Figure 5C). Moreover, we annotated ∼45 VSVpp fusion events in acidic endosomes, but none was detected in pH-neutral vesicles or at the PM. Collectively, these data show that our triple labeling approach enables reliable detection of pH-dependent single virus fusion with endosomes, as depicted in Figure 1A.

We next sought to estimate HIV-1’s residence time in pH-neutral compartments of TZM-bl cells. Toward this goal, triple labeled JRFLpp were prebound to cells in the cold, cells were washed to remove unbound viruses, and virus entry was initiated by shifting the cells to 37 °C (similar to the protocol used in Figure S2A). As infection progressed, a greater fraction of the total EcpH signal was protected from this acidic buffer (Figure S4A–D). Interestingly a significant fraction (>30%) of the EcpH-positive particles at 1 h post infection was not responsive to acidification of the extracellular solution, demonstrating the delivery of this virus into pH-neutral compartments that are potentially permissive to fusion. Together, our results imply that HIV-1 pseudovirus fusion with the plasma membrane and acidified endosomes of TZM-bl cells is disfavored, and that, surprisingly, this virus almost exclusively fuses with pH-neutral endocytic vesicles.

### pH-Neutral Intracellular Compartments Are Primary Sites for HIV-1 Fusion in CD4+ T Lymphocytes

**Figure 6:**
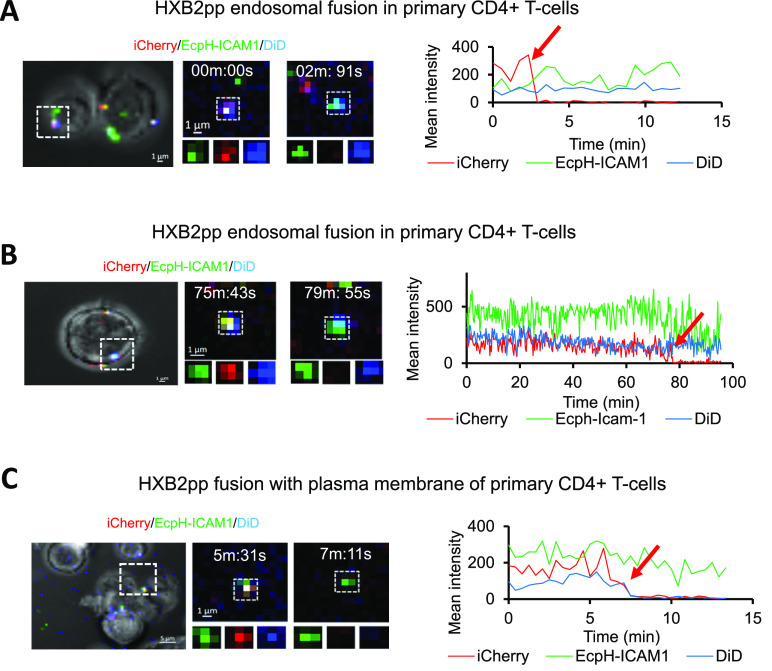
HIV-1 fusion sites in primary CD4+ T-cells. Triple labeled HXB2pp were incubated with activated primary CD4+ T-cells, and live-cell imaging was performed to track single particle fusion. (A, B) Examples of quick (2:34 min, A) and delayed (77:8 min, B) fusion events in pH-neutral vesicles (top left). Respective fluorescence traces (top right) are shown (see Movies S8 and S9). DiD signal was retained at the time of iCherry release for both particles. (C) Time-lapse images (top left) and fluorescence traces (top right) of single HXB2pp fusion with the PM of a primary CD4+ T-cell at ∼7 min is shown. Fusion with the PM is manifested in concomitant loss of iCherry and DiD markers. See Movie S10. Red arrows on fluorescence intensity plots mark virus fusion.

Next, we investigated the sites of HIV-1 fusion in relevant target cells, such as activated primary human CD4+ T-cells and the CEM.NKR-CCR5.Luc T-cell line.^32^ This CEM-derived cell line (hereafter termed CEM.CCR5) stably expresses CCR5 and luciferase under Tat-dependent promoter. Single virus tracking showed that, similar to fusion in TZM-bl cells, HXB2pp primarily fused with endosomes and, less frequently, with the PM of primary human CD4+ T-cells (Figure 6) and CEM.CCR5 cells (Figure S5). Figure 6A and 6B show two examples of HXB2pp fusion with pH-neutral endocytic vesicles. These particles maintained their DiD and EcpH signals after releasing iCherry at ∼3 and ∼78 min after initiation of infection, respectively (Movies S8 and S9). As with TZM-bl cells, HIV-1 fusion with the PM of primary CD4+ T-cells was manifested by a loss of iCherry and DiD signals but retention of the EcpH-ICAM-1 signal (Figure 6C and Movie S10). A similar distribution of endocytic and PM HIV-1 fusion events was observed in CEM.CCR5 cells infected with JRFLpp. The viral particle in Figure S5A (white square) is an example of the endocytic event (Movie S11), while fusion with the PM is shown in Figure S5B (Movie S12). We also observed few hemifusion events where DiD signal was lost but viral content marker (iCherry) was not released (Figure S5C and Movie S13).

**Figure 7:**
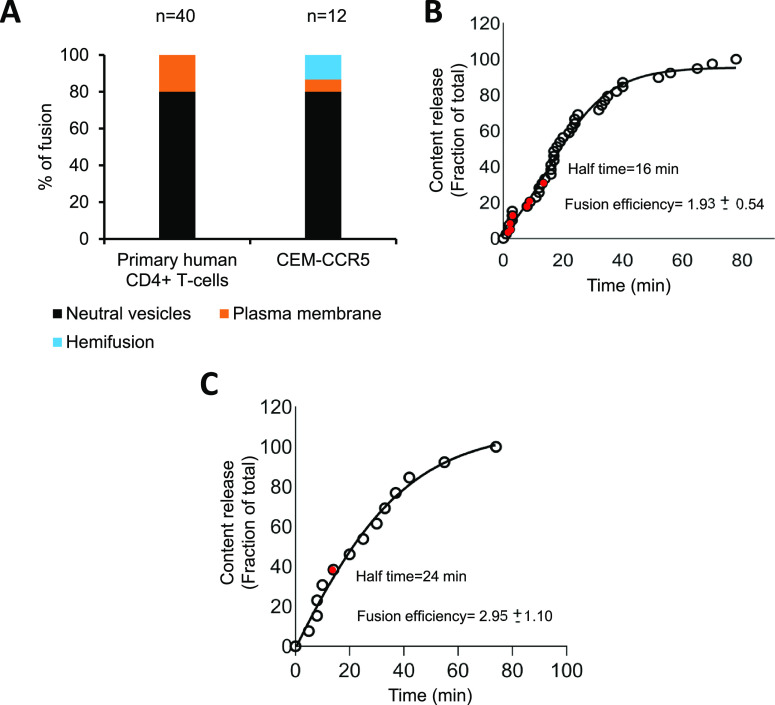
HIV-1 fusion preferentially occurs in pH-neutral endosomes of CD4+ T-lymphocytes. (A) The sites of HIV-1pp fusion in primary CD4+ T-cells and CEM-CCR5, as indicated (*N* is the number of fusion events detected). (B,C) The kinetics of single HIV-1 fusion in primary CD4+ T-cells (B) and CEM-CCR5 cells (C). Red circles mark the PM fusion events. Half-time was measured by sigmoidal curve fitting. HIV-1 Fusion efficiency in primary CD4+ T-cells and CEM-CCR5 are indicated in corresponding graph.

Importantly, HIV-1 pseudoviruses strongly prefer to fuse in pH-neutral intracellular compartments (∼80% events) of CD4+ T-cells, while only a minor fraction of fusion events was detected at the PM (Figure 7A). The half-time for fusion with endosomes of primary human CD4+ T-cells and CEM-CCR5 cells was ∼16 and ∼24 min, respectively (Figure 7B, C), which is faster than in TZM-bl cells (Figure 4B). Pseudovirus fusion with the PM occurred, on average, at ∼10 min (Figure 7B, red circles), indicating that HIV-1 fusion with the PM is relatively rapid compared with fusion with endosomes, perhaps reflecting the need for particle internalization prior to fusion. The fusion efficiency of triple labeled HIV-1pp in both primary CD4+ T-cells and the T-cell line was ∼2–3% (Figure 7B, C, indicated in the graph). Thus, our triple labeling approach reveals a strong preference for HIV-1 fusion with pH-neutral endocytic vesicles of relevant target cells. Importantly, HIV-1 fusion with acidified endosomes was not detected in either T-cell line or primary CD4+ T-cells.

### HIV-1 Infection Is Sensitive to Endocytosis Inhibitors

**Figure 8:**
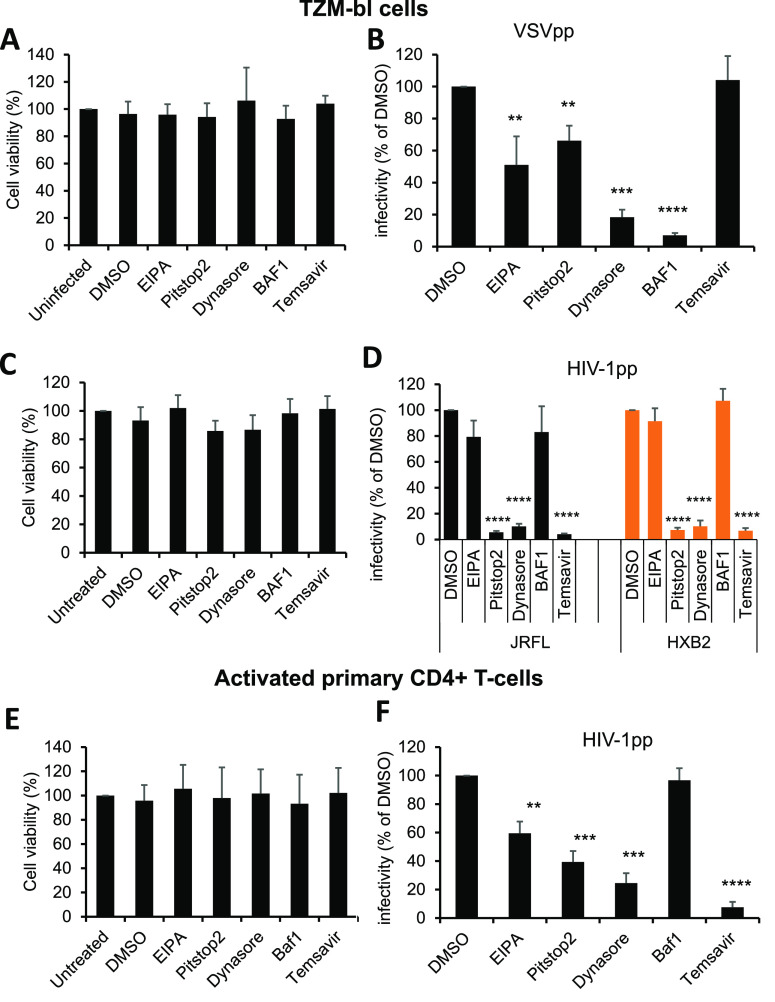
Pharmacological block of endocytosis inhibits HIV-1 infectivity. (A–D) TZM-bl cells were treated either with DMSO or with indicated endocytosis inhibitors EIPA (50 μM), Pistop2 (20 μM), Dynasore (120 μM), Bafilomycin A1 (100 nM) and Temsavir (10 μM). Changes in cell viability were measured by an MTS assay (A,C). The infectivity of triple labeled HXB2pp or VSVpp (B) and JRFLpp (D) in TZM-bl cells is shown. (E,F) Effects of endocytosis inhibitors on cell viability and infectivity of HXB2pp in primary CD4+ T-cells. Data are means ± SD from 3 independent experiments. Statistical analysis done using Student’s *t* test. *, *p* < 0.05, **, *p* < 0.01, ***, *p* < 0.001 and ****, *p* < 0.0001.

Having demonstrated a strong HIV-1 preference for fusion with pH-neutral intracellular compartments, we sought to characterize the endocytic pathway(s) involved in HIV-1 entry. Toward this goal, we used pharmacological inhibitors of distinct endocytic pathways: EIPA (5-[*N*-ethyl-*N*-isopropyl] amiloride, an inhibitor of micropinocytosis),^33^ Pitstop2 (an inhibitor of clathrin mediated endocytosis),^16,34^ and Dynasore (dynamin-2 inhibitor).^35^ First, we verified the expected activity and specificity of EIPA, Pitstop2 and Dynasore using fluorescently tagged dextran known to be internalized via micropinocytosis,^36^ and transferrin that is internalized by clathrin-mediated endocytosis.^37^ EIPA potently inhibited dextran uptake (Figure S6A), while Pitstop2 and Dynasore blocked transferrin uptake in TZM-bl cells (Figure S6B). These results confirm the efficiency of these inhibitors at the concentrations that did not affect cell viability (Figure 8A,C,E).

Next, the effects of the above drugs on VSVpp and HIVpp infectivity in TZM-bl cells were measured using Tat-dependent luciferase expression as readout. VSVpp entry was markedly inhibited by EIPA (∼50%), Dynasore (∼80%) and significantly inhibited by Pitstop2 (∼30%) (Figure 8B), suggesting a mixed route for VSV-G mediated virus entry into TZM-bl cells involving micropinocytosis, dynamin-2 and clathrin-dependent pathway(s). As expected, Temsavir, an HIV-1 entry inhibitor, did not affect the infectivity of VSVpp, which was greatly diminished (>90%) in Bafilomycin A1 (BafA1, vATPase inhibitor) treated cells, confirming the crucial role of low pH in VSV-G-mediated fusion (Figure 8B).

By comparison, EIPA did not significantly impact the infectivity of triple labeled HIVpp in TZM-bl cells, whereas Pitstop2 and Dynasore potently inhibited HIV-1 infection, similar to the specific HIV-1 fusion inhibitor Temsavir (Figure 8D). In control experiments, HIV-1 infectivity was not affected by BafA1, consistent with the pH-independence of HIV-1 Env-mediated membrane fusion.^5^ In contrast to TZM-bl cells, HIV-1 infection of primary human CD4+ T-cells was inhibited by ∼40% with EIPA and by ∼60% and ∼75% with Pitstop2 and Dynasore, respectively (Figure 8F). As expected, Temsavir reduced HIV-1 infectivity by more than 90%, while BafA1 had no impact (Figure 8F). Collectively, these results further support the reliance of HIV-1 on endocytosis to enter and infect susceptible cells.

### Dynamin-2 Is Required for HIV-1 Entry and Infection

**Figure 9:**
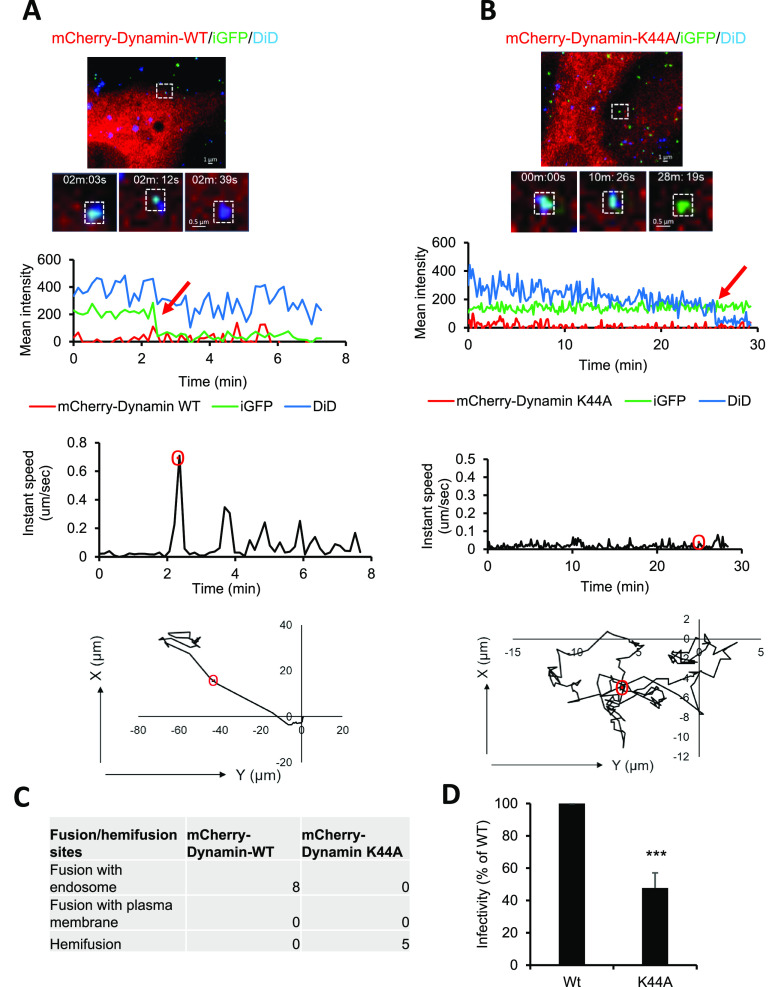
Dynamin-2 plays a role in HIV-1 fusion and infection. (A,B) Double labeled (DiD and Gag-iGFP) JRFLpp were incubated with TZM-bl cells expressing mCherry-Dynamin-2-WT (A) or mCherry-Dynamin-2-K44A (B). (A) Single JRFLpp fusion in endosomes at 2 min 39 s is manifested by loss of GFP but not DiD. (B) Doubled labeled particle undergoes hemifusion (loss of DiD without a loss of the viral content marker). Corresponding fluorescence traces and instant velocities of single JRFLpp hemi/fusion in TZM-bl cells are shown (see Movies S14, S15, S16). (C) Table summarizing the double labeled JRFLpp fusion and hemifusion events in cells expressing WT or K44A dynamin-2 mutant. Four independent live cell imaging experiments were performed, and a total of 13–15 cells were analyzed for single virus fusion for each group. (D) The infectivity of double labeled JRFLpp in TZM-bl cells expressing WT or the K44A dynamin-2 mutant is shown. Data were analyzed by Student’s *t* test. ***, *p* < 0.001. Data are mean ± SEM from an experiment performed in triplicate. Red arrows on fluorescence intensity plots mark virus fusion and hemifusion events.

Although Dynasore blocked the HIV-1 infection in Hela derived cells and CD4+ T-cells, this drug has off target effects.^38^ To confirm the role of dynamin-2 in HIV-1 fusion and infection, we ectopically expressed either mCherry-Dynamin-2-WT or the mCherry-Dynamin-2-K44A mutant in TZM-bl cells. The K44A mutation in dynamin-2, is defective in GTP binding and hydrolysis and dominantly inhibits the dynamin-2-WT function.^39^ We confirmed the effect of the dynamin-2 K44A mutant expression on the endocytosis of transferrin by TZM-bl cells. Compared to cells expressing the WT protein, the Dynamin-2 K44A expression robustly inhibited transferrin uptake (Figure S7). To assess the effect of Dynamin on HIV-1 fusion, TZM-bl cells were transfected with either mCherry-Dynamin-2-WT or the K44A mutant and infected with HIV-1 pseudoviruses colabeled with DiD and Gag-iGFP.^15^ Double labeled viruses were used in these experiments to allocate the Cherry channel for dynamin visualization. We thus swapped Gag-iCherry for Gag-iGFP and did not label pseudoviruses with EcpH-ICAM-1. The justification for sacrificing the EcpH-ICAM-1 pH-sensor comes from the fact that HIV-1 does not fuse with the acidic compartment of cells used in our experiments. The fusion of these double labeled particles was manifested by the loss of iGFP signal.^40^ In cells expressing mCherry-Dynamin-2-WT, the iGFP marker was released without a loss of DiD, implying that fusion occurred in an endocytic compartment (Figure 9A, Movies S14 and S15). Importantly, we were unable to detect the release of the iGFP (viral content marker) but reproducibly saw viral lipid redistribution into the PM manifested by a loss of DiD signal (hemifusion) in mCherry-Dynamin-2 K44A expressing cells (Figure 9B and Movie S16). Moreover, internalization of the virus particle manifested by a surge in the particle’s velocity at the time of fusion was typically observed in Dynamin-2-WT expressing cells but not in Dynamin-2 K44A mutant expressing cells (Figure 9A and B, instant speed plot). A total of 8 endocytic fusion events were detected in control (Dynamin-2-WT expressing) cells, while 5 hemifusion events and no full fusion events were detected in the Dynamin-2 K44A mutant expressing cells (Figure 9C). Thus, blocking dynamin-2 dependent endocytosis allows the initiation but not completion of HIV-1 fusion at the PM. Endocytic HIV-1 entry is likely a productive infectious pathway since HIV-1 infectivity was significantly reduced in cells expressing the Dynamin-2 K44A mutant, as compared to control cells (Figure 9D). The relatively modest reduction in infectivity in these experiments is not unexpected, given the relatively low transfection efficiency of TZM-bl cells (∼30%) with dynamin-2-expressing plasmids. These data support a prominent role of dynamin-2 mediated endocytosis in HIV-1 fusion and infection.

### Plasma Membrane Is a Poor Target for VSV G Protein Mediated Fusion

Having documented poor HIV-1 pseudovirus fusion with the PM, we asked if this phenotype is specific for HIV-1, while other viruses can effectively fuse at the cell surface. To ensure that only fusion with the PM is measured, we prebound to VSVpp to cells in the cold and triggered their fusion by exposure to a pH 5.5 citrate buffer (referred to as “forced fusion”).^41^ Except for control experiments, where VSVpp entry occurred via a conventional endocytic pathway, without exposure to low pH, cells were pretreated with BafA1 to raise endosomal pH. Using this forced fusion protocol, we measured the extent VSVpp fusion with the PM of TZM-bl or lung epithelial A549 cells (Figure S8). Because TZM-bl cells express endogenous levels of interferon-induced transmembrane proteins (IFITMs),^42^ which can inhibit viral fusion, these cells were infected after preincubation in the absence (DMSO) or in the presence of cyclosporine A (CsA) to suppress the IFITMs’ antiviral activity.^43,44^ A549 cells, which express very low levels of IFITMs,^45^ were infected without CsA pretreatment. We found that forced VSVpp fusion with the PM of either TZM-bl or A549 cells was very inefficient, in sharp contrast with viral robust viral fusion through the endocytic pathway (Figure S8A, B). These results support the notion that the PM may not be conducive to viral fusion irrespective of the cellular receptor specificity and fusion triggering mechanism.

## Discussion

Here, we employed an approach to delineate the HIV-1 entry sites into target cells that involves labeling the HIV-1 particles with three fluorescent probes reporting the viral content and lipid release into the cell upon fusion as well as the changes of the extraviral pH prior to fusion. This virus labeling approach combined with single particle tracking reveals that, surprisingly, HIV-1 predominantly fuses with pH-neutral endocytic vesicles in Hela-derived cells (∼90%), the CD4+ T-cell line (∼80%), and primary human CD4+ T-cells (∼80%). Only a small fraction of pseudoviruses (∼10–20%) fuse or hemifuse with the PM of these cells. Moreover, all intracellular fusion events exclusively occurred in pH-neutral vesicles and never in acidified endosomes, in stark contrast to VSV-G mediated fusion. Several studies, including ours, support the notion that productive HIV-1 entry occurs via an endosomal pathway. Potent inhibition of HIV-1 infection by Pitstop2 and Dynasore in TZM-bl cells and primary CD4+ T-cells (Figure 8) supports the role of clathrin and dynamin-2 in productive HIV-1 entry. However, in contrast to TZM-bl cells, infection of primary CD4+ T-cells is significantly inhibited by the micropinocytosis inhibitor, EIPA, indicating the existence of multiple endocytic routes for HIV-1 entry into these cells.

We interpreted loss of iCherry without reduction of DiD or EcpH signals (Figures 2, 3 and 6) as HIV-1 fusion in pH-neutral endosomes. An alternative explanation for this result is virus fusion with endocytic vesicles still connected to the PM (prior to budding off the PM) that somehow restrict DiD diffusion to the PM. Two lines of evidence argue against this possibility. First, overexpression of dominant-negative dynamin-2 mutant inhibits HIV-1 pseudovirus fusion (iCherry release), while allowing lipid mixing at the PM (Figure 9). Second, application of membrane-impermeant acidic buffer after varied times postinfection does not affect the EcpH signal of a fraction of pseudoviruses (Figure S4), implying that these particles reside in pH-neutral compartments that are not connected to extracellular space.

The role of dynamin-2 in HIV-1 infection and fusion has been investigated in several studies.^46−48^ Previously, we have shown that Dynasore, a dynamin-2 inhibitor, abolishes the viral content release but permitted most HIV-1 particles to transfer their lipids to the PM.^15^ In agreement with these results, we now demonstrate that the dominant-negative K44A mutant of dynamin-2 inhibits HIV-cell fusion but favors the transfer of a lipid dye from the virus to the PM (hemifusion). Our data imply that HIV-1 does not efficiently fuse at the surface of diverse cell lines and primary cells and that viral fusion with the PM does not progress beyond the lipid mixing stage when the dynamin-2 function (and likely virus endocytosis) is impaired. This notion is supported by the results in Figure S8, demonstrating poor efficiency of low pH-driven (forced) VSVpp fusion with the plasma membrane of TZM-bl and A549 cells.

It should be noted that an earlier study by our group^24^ employed a similar pseudovirus labeling strategy with EcpH-ICAM1 and Gag-iCherry (without DiD) but did not detect viral fusion at neutral pH. Although viral content release has been observed in a very small fraction of particles, these events were not blocked by HIV-1 fusion inhibitors and have therefore been interpreted as spontaneous virolysis. The increased rate of spontaneous virolysis suggested that the prior labeling protocol caused viral membrane instability. Here, we made a few changes to the virus labeling protocol. First, we used a higher pR9ΔEnv/Gag-iCherry plasmid ratio, which we find to be less impactful for infectivity. Second, and more importantly, we reduced the EcpH-ICAM1 to HIV-1 backbone plasmid ratio by 3-fold. This labeling protocol enabled reliable identification of HIV-1 pseudovirus fusion at neutral pH, which was sensitive to fusion inhibitors. We therefore surmise that excessive incorporation of EcpH-ICAM1 into pseudovirions diminishes their fusion competence or destabilizes the viral membrane. Inclusion of DiD in the current study does not noticeably affect the virus’ infectivity or fusion capacity but may further reduce the probability of spontaneous virolysis.

What are the reasons for the strong HIV-1 preference for fusion with pH-neutral intracellular compartments? This phenotype could result from a kinetic competition between HIV-1 fusion and uptake by cells^3^ so early fusion events can occur on the cell surface, whereas delayed fusion takes place in endosomes. In other words, the relative rates of HIV-1 fusion and endocytosis in a given cell type can modulate the entry site. Indeed, we found that the half-time of HIV-1 fusion with TZM-bl and T-cells cells is ∼40 and ∼20 min, respectively. In comparison, fusion with PM usually occurred within ∼3–10 min. However, our attempts to redirect HIV-1 fusion to the PM by synchronizing, and thereby accelerating HIV-1 fusion, or by inhibiting endocytosis (Figure 9 and^49^) were unsuccessful. These results suggest that HIV-1 fusion with the PM of TZM-bl cells and CD4+ T-cells is disfavored beyond the kinetic competition between fusion and endocytosis. Thus, the striking preference for HIV-1 fusion within neutral intracellular compartments (Figures 4 and 7) and its reliance on endocytic trafficking factors^21^ cannot be readily explained by quick virus uptake. Moreover, quick endocytosis does not explain the lack of fusion of HIV-1 with acidified endosomes. We also found that, surprisingly, a significant fraction of HIV-1pp appears to accumulate in pH-neutral intracellular compartments of TZM-bl cells that are not connected to the extracellular milieu. These virus-harboring vesicles could be the primary sites of HIV-1 fusion. It is currently unclear what disfavors HIV-1 fusion with PM or acidified endosomes and what makes pH-neutral vesicles conducive to fusion.

Entry through the endocytic route may have advantages for HIV-1. For instance, this pathway may allow the incoming viral cores to bypass the subcortical actin cytoskeleton, which may be a major barrier for the viral core transport to the cytoplasm and into the nucleus.^50^ In addition, virus clearance from the cell surface may protect it from neutralizing antibodies and inhibitors targeting intermediate conformations of Env. Indeed, we have previously shown that delayed HIV-1 uptake increases the potency of the inhibitory C34 peptide and neutralizing antibodies.^15,51^ Future studies aimed at characterizing the compartments permissive for fusion will shed light on the yet unknown factors and processes that facilitate HIV-1 fusion with nascent endocytic vesicles.

### Limitations of This Study

While the virus labeling and tracking techniques used in this study reveal previously unappreciated sites of HIV-1 entry, these approaches are not without limitations. One issue is that not all pseudovirions are labeled with three fluorescent markers (viral lipid, viral content, and an extra-viral pH sensor). Typically, less than 70% of pseudoviruses produced by our protocol are triple labeled, so the fusion sites of a considerable fraction of particles cannot be unambiguously determined. However, given that the specific infectivity of triple labeled pseudoviruses is not significantly compromised, it seems fair to assume that the fusion sites of triple labeled pseudovirions are representative of those of unlabeled HIV-1. Another limitation relates to the use of pseudoviruses in lieu of infectious HIV-1 particles. Further improvements in virus labeling techniques, including the labeling of replication competent HIV-1, would help to better delineate the entry pathways of this virus.

## Conclusions

A combination of single HIV-1 pseudovirus tracking in living cells and labeling distinct viral components employed in this study provides critical insights into the virus’s entry pathways in different cell types, including primary human CD4+ T-cells. We found that, surprisingly, HIV-1 selectively fuses with pH-neutral intracellular vesicles and, much less frequently, with the plasma membrane. Interestingly, HIV-1 fusion with acidified endosomes could not be detected. Attempts to redirect HIV-1 fusion to the plasma membrane by blocking virus uptake promoted hemifusion, but not full fusion. Our findings imply that HIV-1 fusion with the plasma membrane is disfavored and that, in contrast, early endocytic vesicles are conducive to viral fusion. Understanding the basis for a strong HIV-1 preference for fusion with pH-neutral intracellular vesicles may reveal additional targets for therapeutic interventions.

## Materials and Methods

### Cell Lines, Reagents, and Plasmids

TZM-bl cells ectopically expressing CD4 and CCR5^31^ and CEM-NKR-CCR5-Luc cells^32^ were from the NIH AIDS Reagent Program. HEK293T/17 (human embryonic kidney) cells were purchased from ATCC (Manassas, VA, USA). TZM-bl and HEK293T/17 cells were grown in Dulbecco’s Modified Eagle Medium high-glucose (DMEM, Cellgro, Manassas, VA). CEM-NKR-CCR5-Luc cells were grown in an RPMI-1640 (Cellgro). For all cells, the medium was supplemented with 10% heat-inactivated Fetal Bovine Serum (FBS, HyClone Laboratories, Logan, UT) and 100 U penicillin-streptomycin (Gemini Bio-Products, West Sacramento, CA). For HEK293T/17 cells, the growth medium was supplemented with 0.5 mg/mL G418 sulfate (Life Technologies, Inc., Grand Island, USA). Human primary peripheral blood mononuclear cells (PBMCs) were purified from the peripheral blood of healthy donor (approval from Emory “Phlebotomy of Healthy Adults for Research in Infectious Diseases and Immunology”) by Ficoll-Paque Plus (GE Healthcare, Piscataway, NJ) density gradient centrifugation. CD4^+^ T lymphocytes were isolated from PBMCs by depletion of non-CD4^+^ cells (negative selection) using MACS CD4+ T Cell Isolation kit II (Miltenyi Biotec, Auburn, CA) and MACS LD columns (Miltenyi Biotec, Auburn, CA). Purified CD4^+^ T cells were cultured in RPMI-1640 medium supplemented with 10% heat-inactivated FBS, 10 ng/mL of Interleukin-2 (NIH AIDS Reagent Program), and 2.5 μg/mL of phytohemagglutinin P (Sigma, St. Louis, MO) to activate the cells. Cells were used for the fusion/infectivity experiments 3 days postactivation. FluoroBrite DMEM was obtained from Life Technologies and used for incubating the cells during live cell imaging for virus-cell fusion.

The pCAGGS plasmid encoding HXB2 or JRFL Env was provided by J. Binley (Torrey Pines Institute). The pMDG-VSV-G plasmid expressing VSV-G was a gift from J. Young (Roche). The HIV-1-based packaging vector pR9ΔEnv was obtained from Dr. C. Aiken (Vanderbilt University). The HIV-1 Gag-iCherryΔEnv, the ecliptic pHlourin-ICAM-1 (EcpH-ICAM) chimera,^23^ mCherry-Dynamin-2 WT and K44A mutant expressing vectors have been described previously.^15^ DiD was purchased from ThermoFisher Scientific (D7757). Temsavir (BMS-529) was obtained from Aurum Pharmatech (W-5969). Bafilomycin A1 (B1793), Dynasore (D7693), Pitstop2 (SML1169) and NH_4_Cl (A0171) were from Sigma. The C34 peptide was from NIH HIV Reagents Program (Cat# ARP-9824). EIPA was obtained from Tocris (3378).

### Pseudovirus Production and Infectivity Assays

HEK293T/17 cells cultured in a 10 cm dish were transfected with the following amounts of plasmids: 4 μg of pR9ΔEnv, 2 μg of Gag-iCherryΔEnv, 0.5 μg of pcRev, 2 μg of EcpH-ICAM1 and 1.5 μg of HXB2 or JRFL Env plasmids. Transfection was carried out using JetPrime transfection reagent (purchased from Polyplus cat#114–15). The pcDNA (empty) vector was used in place of Gag-iCherry/EcpH-ICAM1 to produce unlabeled viral particles. For generation of triple labeled VSV-G pseudotyped HIV-1 particles, 0.5 μg of VSV-G was used instead of HIV-1 Env-encoding plasmids. DiD and Gag-iGFP based double labeled HIV-1 particles were generated, as described previously.^15^ The p24/Gag content of the virus stock was measured by p24 enzyme-linked immunosorbent assay (ELISA) as described previously.^52^

For virus infectivity, TZM-bl in a clear bottom 96-well black plate was washed with PBS 2 times and fresh media was added. Later, triple labeled HIVpp or VSVpp were bound to cells by centrifugation at 4 °C for 30 min at 1550*g* with serial dilution. Plates were placed in 37 °C incubator for 36 h postinfection, and then cells were incubated with 50 μL of Bright-Glo luciferase substrate (Promega, Madison, WI) at room temperature for 5 min, and the luciferase activity was measured by a TopCount NXT plate reader (PerkinElmer Life Sciences, Waltham, MA, USA).

To assess the infectivity of HIV-1 particles in primary human CD4+ cells, we generated luciferase reporter virus particles by transfecting HEK293T/17 cells with 4 μg of HIV-1-based packaging vector NL4-3-E-R-Luc ((NIH AIDS Reagent Program), 0.5 μg of pcRev, 2 μg EcpH-ICAM1 and 1.5 μg of HXB2 in 10 cm dish. Twelve hours post-transfection, the medium was exchanged to fresh serum-free DMEM containing 30 μM DiD dye, and incubated for 4 h at 37 °C, after which time, the medium was replaced with phenol red-free DMEM containing 10% FBS. 48 h post-transfection, the supernatant was collected, aliquoted, and stored at −80 °C.

For virus infectivity in the presence of endocytic inhibitors, TZM-bl cells in a clear-bottom 96-well black plate or primary CD4+ T-cells in round-bottom 96-well plates were washed with PBS two times to remove serum containing medium. Later, cells were pretreated with following pharmacological inhibitors or DMSO, as indicated, for 30 min in serum free medium: Dynasore (120 μM); Pitstop2 (20 μM); EIPA (50 μM); Bafilomycin A1 or Temsavir (10 μm). After preincubation with endocytic inhibitors, triple labeled HIVpp or VSVpp were bound to cells by centrifugation at 4 °C for 30 min at 1550*g* (MOI = 1). Plates were placed in 37 °C incubator for 2 h to allow fusion, after which time, medium was replaced with fresh DMEM supplemented with 10% FBS. 36 h postinfection, luciferase activity was measured as mentioned above.

Before the luciferase signal was read, the MTS substrate (CellTiter Aqueous One; Promega) was added to the wells. Plates were incubated for 30 min at 37 °C and 5% CO_2_, and cell viability was measured by absorbance at 490 nm (Synergy HT plate reader).

### Western Blotting

For Western blot analysis, 10 pg of p24/well was loaded onto a polyacrylamide 4–15% gradient gel (Bio-Rad). Proteins were transferred onto a nitrocellulose membrane, blocked with 10% blotting-grade blocker (Bio-Rad) for 30 min at room temperature, and incubated with human anti-HIV serum (HIV IgG) (NIH HIV Reagent Program) (1:2000 dilution) for 1 h at room temperature. Blots were washed three times after 10 min intervals with PBST (PBS + 0.1% Tween). Secondary antibody IRDye 800CW Goat anti-Human IgG antibody from Li-Cor (926-32232) was used. Precision Plus Protein Standards (Kaleidoscope, Bio-Rad) were used as molecular weight markers. The blots were imaged on an LI-COR (Odyssey CLx).

### Single-Virus Imaging in Live and Fixed Cells

Triple labeled pseudoparticles were immobilized on poly-l-lysine–coated 8-well chamber coverslips (Lab-Tek, Nalge Nunc International, Penfield, NY) for 30 min at 4 °C, and wells were washed with cold Ca^2+^/Mg^2+^-containing phosphate buffer (PBS^++^) to remove unbound virus. Virus samples were imaged before and after the addition of 0.1 mg/mL of saponin to permeabilize the viral membrane and release the iCherry content marker. Single-virus fusion experiments were performed with either TZM-bl, CEM-CCR5 or primary human CD4+ T-Cells. Cells were plated on collagen-coated glass-bottom dishes (MatTek, Ashland, MA) in FluoroBrite DMEM/10% FBS and grown to 70% confluency. Before imaging, the cells were chilled on ice for 3–5 min, washed with ice-cold PBS^++^, and spinoculated with freshly thawed pseudovirus for 30 min at 1550*g* at 4 °C. After centrifugation, cells were washed with ice-cold PBS^++^ to remove unbound virus. Virus-cell fusion was initiated by the addition of a prewarmed live-cell imaging buffer that contained 20 mM HEPES and 1× Glutamine. Images were acquired with a Zeiss LSM880 laser scanning confocal microscope equipped with an environmental chamber to maintain 37 °C and humidity and a DefiniteFocus module. Samples were imaged using a C-Apo 40×/1.2NA water-immersion objective or a 63*×*/1.4NA oil-immersion objective. A field of view was selected, and full cell volume was imaged by acquiring 8–12 Z-stacks spaced by 0.7 μm every 18–35 s. Acquired image series were converted to maximum intensity projections and analyzed. Live cell imaging was done for either 60 min (VSV-G) or 120 min (HXB2 or JRFL). EcpH, mCherry, and DiD fluorescence were excited using highly attenuated 488, 561, and 633 nm laser lines, respectively. For fixed cell imaging, samples were treated with 4% paraformaldehyde for 30 min at room temperature. Image stacks with a Z interval of 0.6 μm were acquired and represented by a maximum intensity projection.

### Virus Uptake Assay

TZM-bl cells were seeded in collagen-coated glass-bottom imaging dishes. JRFLpp were prebound to cells by centrifugation at 4 °C for 30 min at 1550*g*. Cells were washed with cold buffer to remove unbound viruses, and one dish was imaged immediately (0 min) in phosphate buffer using 2 × 2 tile scan with 10–12 Z-stacks. An acidic pH 5.0 citrate buffer (50 mM citrate buffer, 5 mM KCl, 2 mM CaCl_2_, 90 mM NaCl) was added to the imaging dish in >25-fold excess relative to phosphate buffer, and the same fields were imaged using identical settings. Cells were shifted to 37 °C (with complete media) and the above imaging and citrate buffer application steps were repeated after washing the cells two times with Phosphate buffer at indicated times. Images (maximum intensity projections) were analyzed using ImageJ to measure the fraction of triple labeled particles that retained the EcpH signal over time and the fraction of EcpH+ viruses that did not respond to a low pH application.

### Image Analysis and Single-Particle Tracking

The acquired time-lapse Z-stack images were converted to maximum intensity projections for single particle tracking. Single fusion events were annotated using the ImageJ ROI manager tool. The times of iCherry and/or DiD loss, which occurred in a single image frame, were determined visually as the time of color change. Representative single virus fusion events were tracked using ICY image analysis software (icy.bioimageanalysis.org). The labeled pseudoviruses were identified by the Spot Detection plugin and tracked using the Spot Tracking plugin to determine the fluorescence intensity over time, particle trajectory, and instant velocity.

### Endocytosis Inhibition by Pharmacological Drugs

For dextran uptake assay, TZM-bl cells were preincubated with DMSO or EIPA (50 μM) for 30 min. We added 150 μg/mL tetramethylrhodamine dextran (TMR-dextran, ThermoFisher Scientific, D1819, 70,000 MW) to cells and incubated at 37 °C for 30 min.

For transferrin uptake measurements, TZM-bl cells were pretreated with Dynasore (120 μM), Pitstop2 (20 μM) or DMSO (control) in serum-free medium. Cells were kept on ice for 5 min, and Transferrin-fluorescein (Transferrin from Human Serum, Fluorescein Conjugate, ThermoFisher Scientific, T2871, 50 μg/mL) was added and incubated on ice for 15 min. Unbound transferrin was removed by two PBS washes, and the cells were placed at 37 °C for 10 min. EIPA, Pitstop2 or Dynasore were maintained in medium throughout the experiment (during preincubation, washing, and postincubation). Cells were washed with PBS and fixed with 4% paraformaldehyde at 37 °C. Wheat Germ Agglutinin (WGA) Alexa Fluor 633 Conjugate (ThermoFisher Scientific, W21404) was used to label the cell membrane. Fluorescence intensity was measured using a 488 nm laser line for transferrin-fluorescein, a 561 nm laser line for Dextran-TMR, and a 633 nm laser line for WGA imaging.

### Statistical Analysis

Unless otherwise noted, all bulk experiments were carried out in duplicates and repeated at least three times. On average, 5–15 independent single-virus imaging experiments were performed for each condition. Statistical analysis was carried out between groups using Student’s *t* test using GraphPad Prism 10, as stated in the figure legends. *p* < 0.05 were considered significant.

## Supporting Information Available

Figures S1–S7: Supplementary figures provide additional information related to validation of the virus labeling approach and control experiments (PDF)
Movie S1: JRFLpp fusion event with pH-neutral vesicles in TZM-bl cell (MP4)
Movie S2: JRFLpp fusion with the plasma membrane of TZM-bl cell (MP4)
Movie S3: JRFLpp hemifusion with the plasma membrane of TZM-bl cell (MP4)
Movie S4: HXB2pp fusion with pH-neutral vesicles in TZM-bl cell (MP4)
Movie S5: HXB2pp fusion with the plasma membrane of TZM-bl cell (MP4)
Movie S6: JRFLpp entry into acidified endosome of TZM-bl cell without fusion (MP4)
Movie S7: VSVpp fusion in acidified endosome of TZM-bl cell (MP4)
Movie S8: HXB2pp fusion events with pH-neutral vesicles in primary human CD4+ T-cell (MP4)
Movie S9: HXB2pp fusion events with pH-neutral vesicles in primary human CD4+ T-cell (MP4)
Movie S10: HXB2pp fusion with the plasma membrane of primary human CD4+ T-cell (MP4)
Movie S11: JRFLpp fusion with a pH-neutral vesicle in CEM-CCR5 cell (MP4)
Movie S12: JRFLpp fusion with the plasma membrane of CEM-CCR5 cell (MP4)
Movie S13: JRFLpp hemifusion in the plasma membrane of CEM-CCR5 cell (MP4)
Movie S14: JRFLpp fusion with endosomes in TZM-bl cells expressing iCherry- Dynamin-2 WT (MP4)
Movie S15: JRFLpp fusion with endosomes in TZM-bl cells expressing iCherry- Dynamin-2 WT (MP4)
Movie S16: JRFLpp hemifusion with TZM-bl cell expressing the dominant-negative dynamin2 mutant, iCherry-Dynamin-2 K44A (MP4)
