# Supplementary material for: Human Immunodeficiency Virus 1 Preferentially Fuses with pH-Neutral Endocytic Vesicles in Cell Lines and Human Primary CD4+ T-Cells
Source: ACS Nano. 2023 Aug 17;17(17):17436–50. doi: 10.1021/acsnano.3c05508 (PMC10510587; doi:10.1021/acsnano.3c05508)
Supplement: Supplementary file 1 — nn3c05508_si_001.pdf [file nn3c05508_si_001.pdf]

Supporting Information

Human Immunodeficiency Virus 1 Preferentially Fuses with pH-Neutral Endocytic Vesicles in Cell Lines and Human Primary CD4+ T-Cells

Manish Sharma[1,2], Mariana Marin[1,2], Hui Wu[1], David Prikryl[1], and Gregory B. Melikyan[1,2,*]

[1]Department of Pediatrics, Division of Infectious Diseases, Emory University School of Medicine, Atlanta, Georgia 30322, USA.

[2]Children's Healthcare of Atlanta, Atlanta, Georgia 30322, USA.

Supplementary Figures and Legends

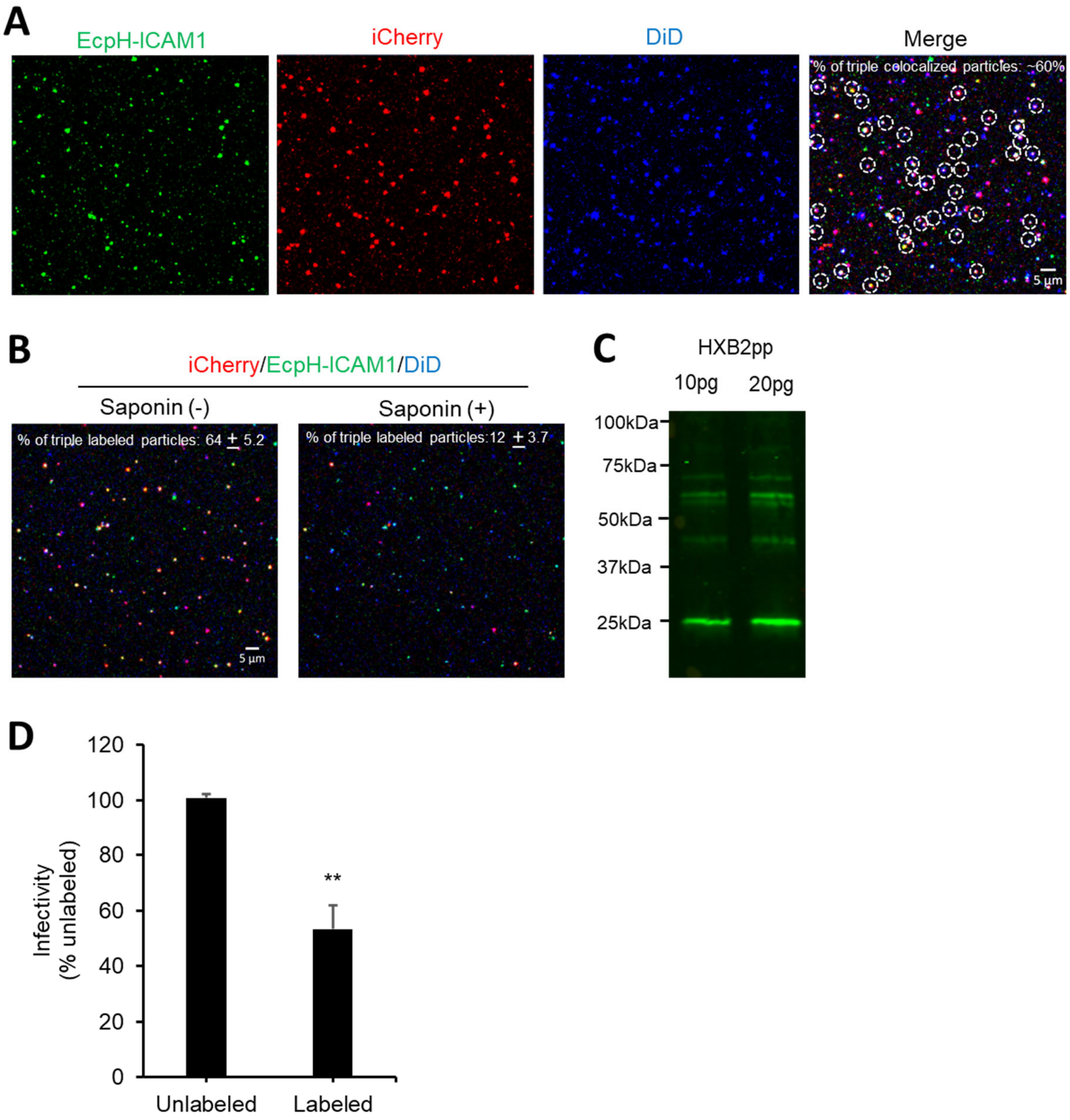

Fig. S1. Characterization of triple labeled HXB2pp. (A) Images of coverslip attached HIVpp pseudotyped with HXB2. Pseudoviruses were co-labeled with iCherry, EcpH-ICAM1, and DiD. White dashed circles represent single virus particles positive for triple fluorescence. (B) Coverslip attached triple single virus particles before and after lysis by saponin (100 µg/mL). (C) p24 Western blot shows Gag processing upon maturation of triple labeled HXB2pp (D) Infectivity of unlabeled and labeled (iCherry, EcpH-ICAM1 and DiD) HXB2pp in TZM-bl cells. Data were analyzed by Student's t-test. **, $p<.01$. Data are mean and SD of two different viral stocks and each experiment performed in triplicate.

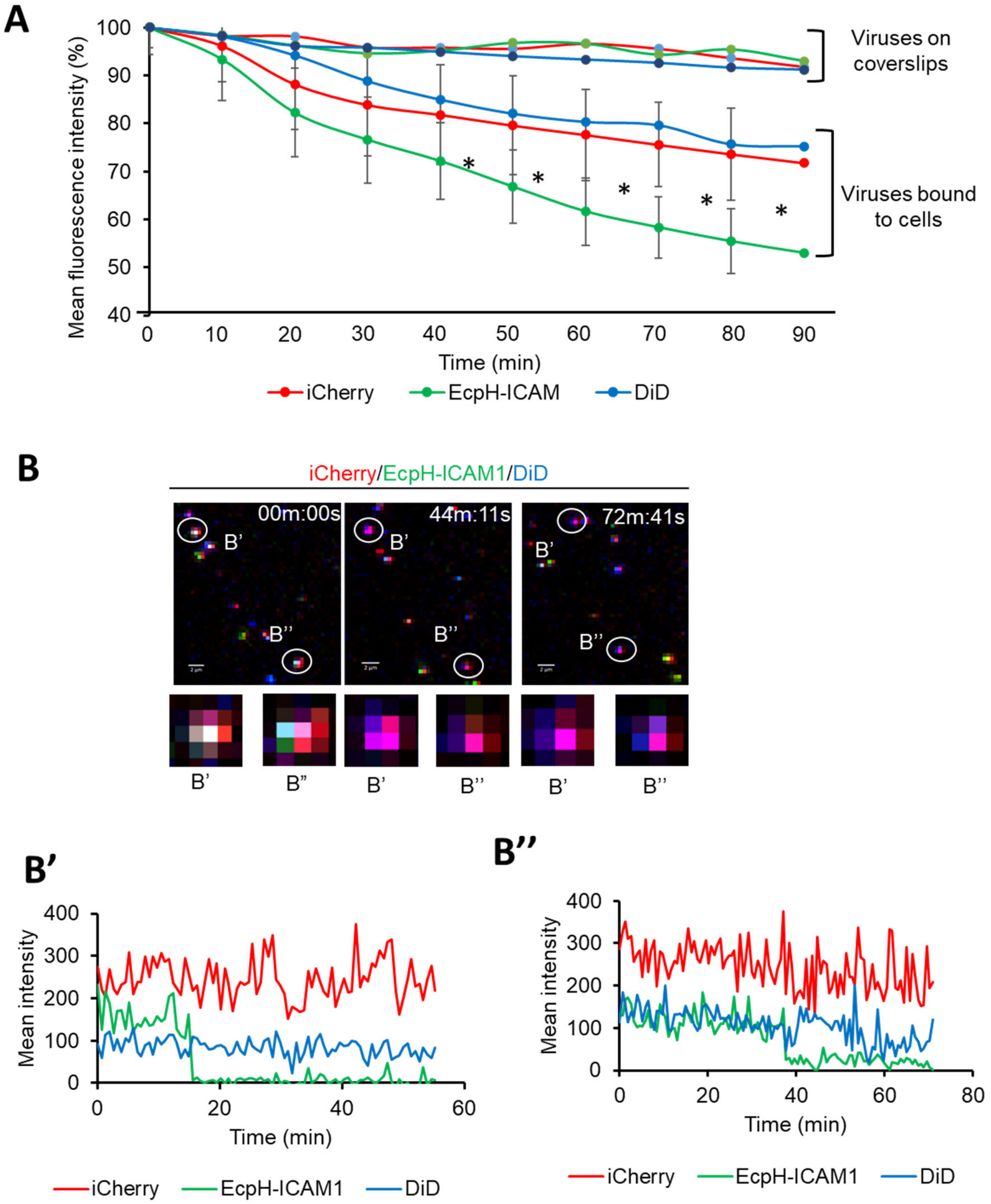

Fig. S2. HIV-1 enters acidic compartments without fusion. (A) The mean fluorescence intensity of EcpH, iCherry and DiD was calculated from triple labeled viruses bound to coverslip or cells and values were plotted as an average of n=4 independent experiments. (B) Representative confocal images of triple labeled particles bound to TZM-bl cells at different time points. Magnified images of two triple labeled particles, annotated by white circle (labeled as B' or B'') were shown in panel B and their respective mean fluorescence intensity of EcpH, iCherry and DiD is shown in panel B' and B'' (Movie 7).

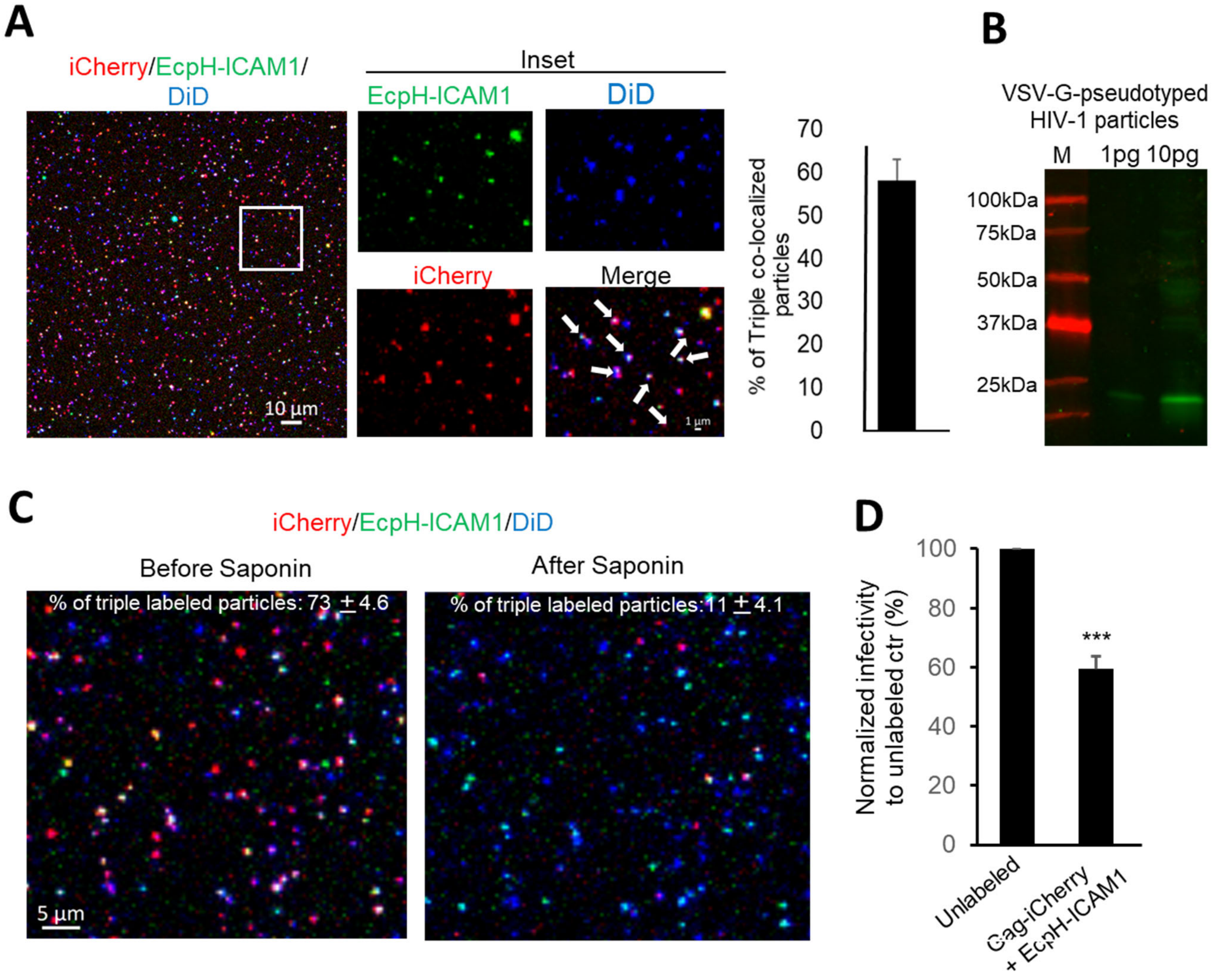

Fig. S3. Characterization of triple labeled VSV-G pseudotyped HIV-1 particles. (A) Confocal image of VSV-G pseudovirus particles attached to a coverslip. Inset shows zoomed-in of the boxed area. White arrows mark triple labeled particles. Bar graph shows the co-localization of three fluorescent markers in viral particles. A total of 493 particles from 3 different image fields were analyzed. (B) Maturation of triple labeled VSV-G pseudoviruses assessed by Western blotting for HIV-1 p24. (C) Infectivity of unlabeled (control) and labeled (iCherry, EcpH-ICAM1, DiD) viral particles in TZM-bl cells. Data are means and SEM from an experiment performed in triplicate.

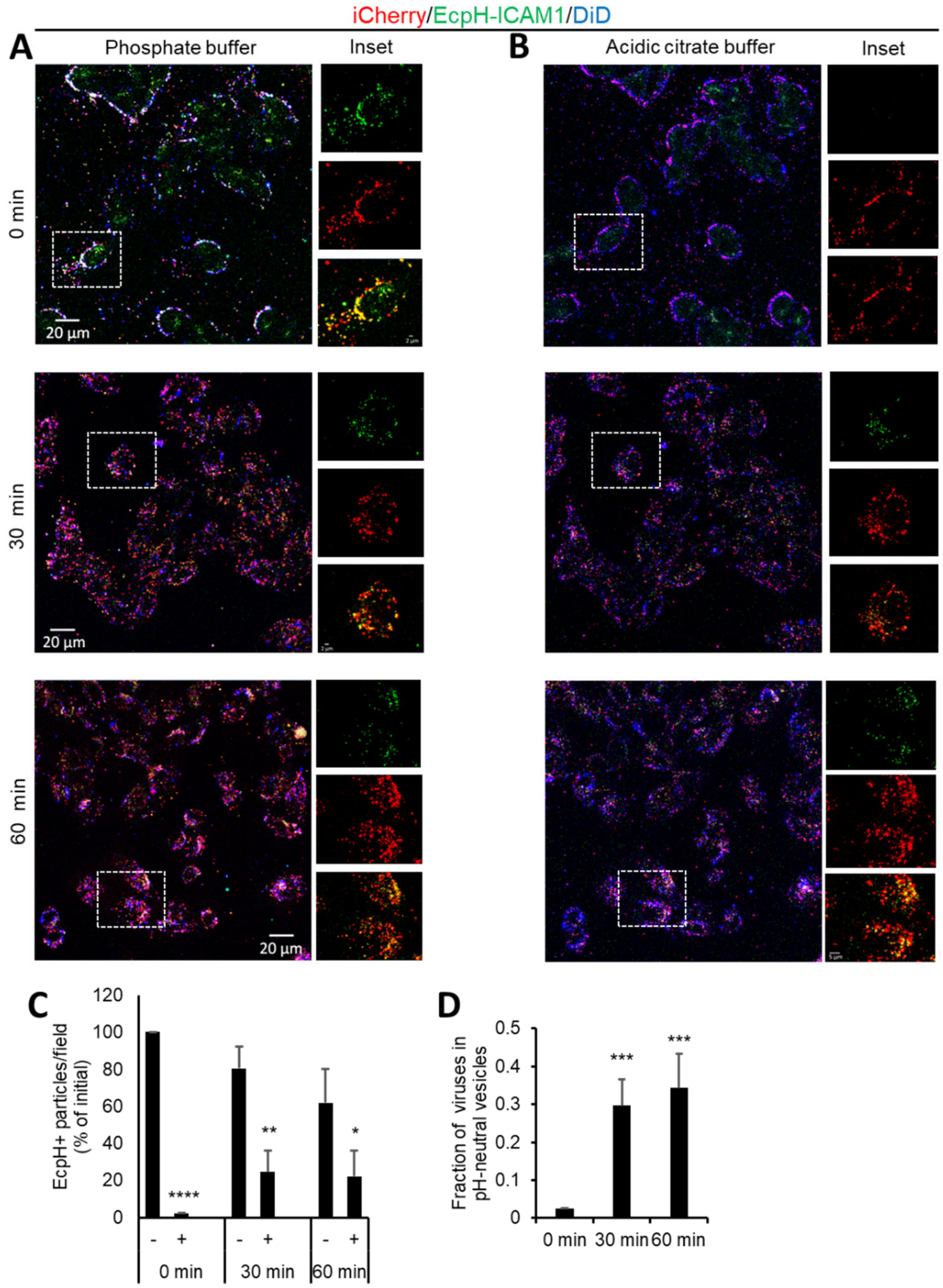

Fig. S4. Uptake of triple labeled JRFLpp in TZM-bl cells. Representative confocal images of TZM-bl cells infected with JRFLpp labeled with iCherry (red), EcpH-ICAM1 (green) and DiD (blue). Cells were exposed to (A) phosphate buffer, pH 7.2 or (B) membrane impermeable acidic buffer (citrate buffer pH 5.0) at 0, 30 and 60 min. Magnified regions from the boxed areas are shown in corresponding insets. (C) The time course of virus uptake and entry into acidified endosomes plotted as the ratio of EcpH and iCherry signals in presence or absence of citrate buffer at indicated time points. (D) The fraction of normalized EcpH signal that does not respond to application of acidic buffer. Data are mean and SD of two different experiment and total 8 imaging fields analyzed by Student's t-test. *, p<.05, **, p<.01, ***, p<.001 and ****, p<.0001.

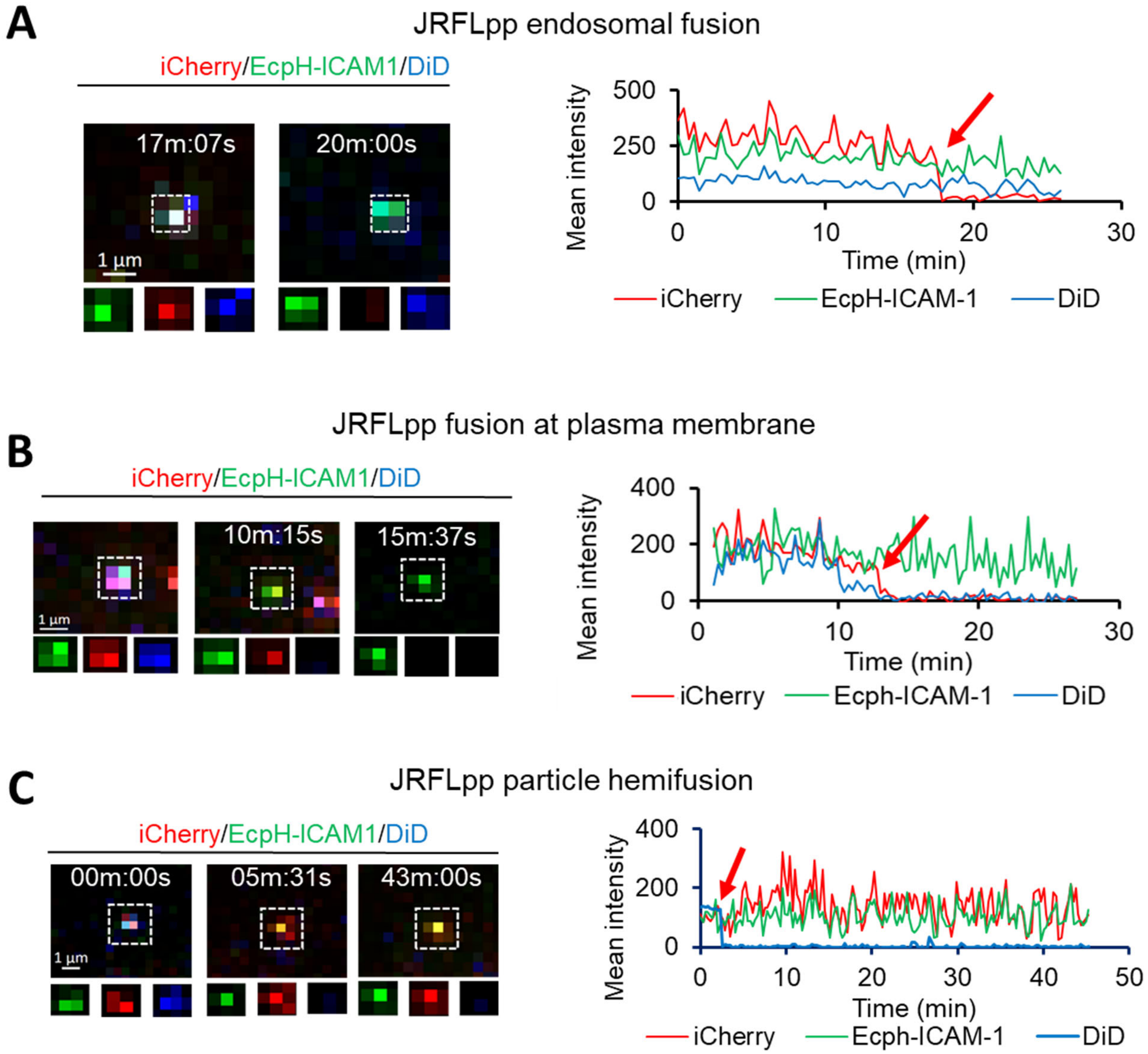

Fig. S5. Fusion sites of JRFLpp in CEM-CCR5 cells. (A, B) Triple labeled JRFLpp fusion in the pH-neutral endocytic vesicle and with the PM, respectively. In panels A and B, time-lapse images (top left), fluorescence traces (top right). Endosomal fusion in panel A is manifested by iCherry loss at 18:2 min without DiD or EcpH signal loss, whereas fusion with the PM (panel B) detected at 12:76 min is associated with a simultaneous loss of iCherry and DiD, without loss of EcpH (see Movies 11 and 12). (C) JRFLpp hemifusion event (loss of DiD without loss of iCherry) (see movie 13). Red arrows on fluorescence intensity plots mark virus fusion and hemifusion events.

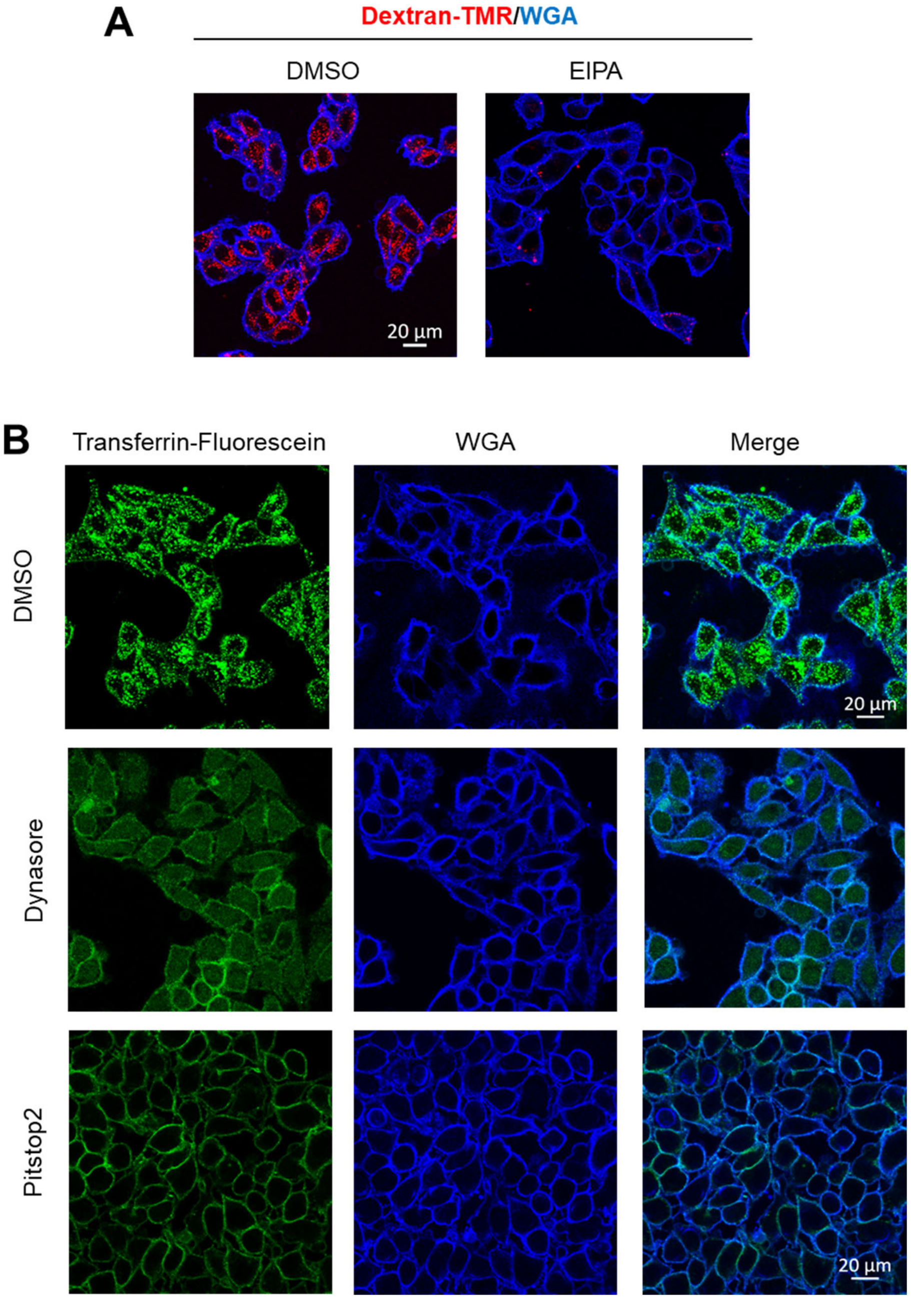

Fig. S6. Validation of endocytosis inhibitors. (A) Representative confocal images showing uptake of Dextran-TMR (red) by TZM-bl cells in presence of DMSO (control) and EIPA (50 µM). (B) Confocal images showing uptake of fluorescein-tagged transferrin by TZM-bl cells in presence of DMSO and Pitstop2 (20 µM) or Dynasore (120 µM). Cells were pre-treated with indicated inhibitors in a serum-free medium for 30 min. For the dextran uptake assay, TMR-Dextran was added to media and incubated at 37 ºC for 30 min. For the transferrin uptake assay, transferrin-fluorescein was added to cells on ice for 10 min. Cells were washed and incubated at 37 ºC for 10 min. Inhibitors were maintained throughout the experiment (pre-incubation, washing and chase). Cells were fixed with 4% PFA, and PM was labeled with Wheat germ agglutin (WGA-633).

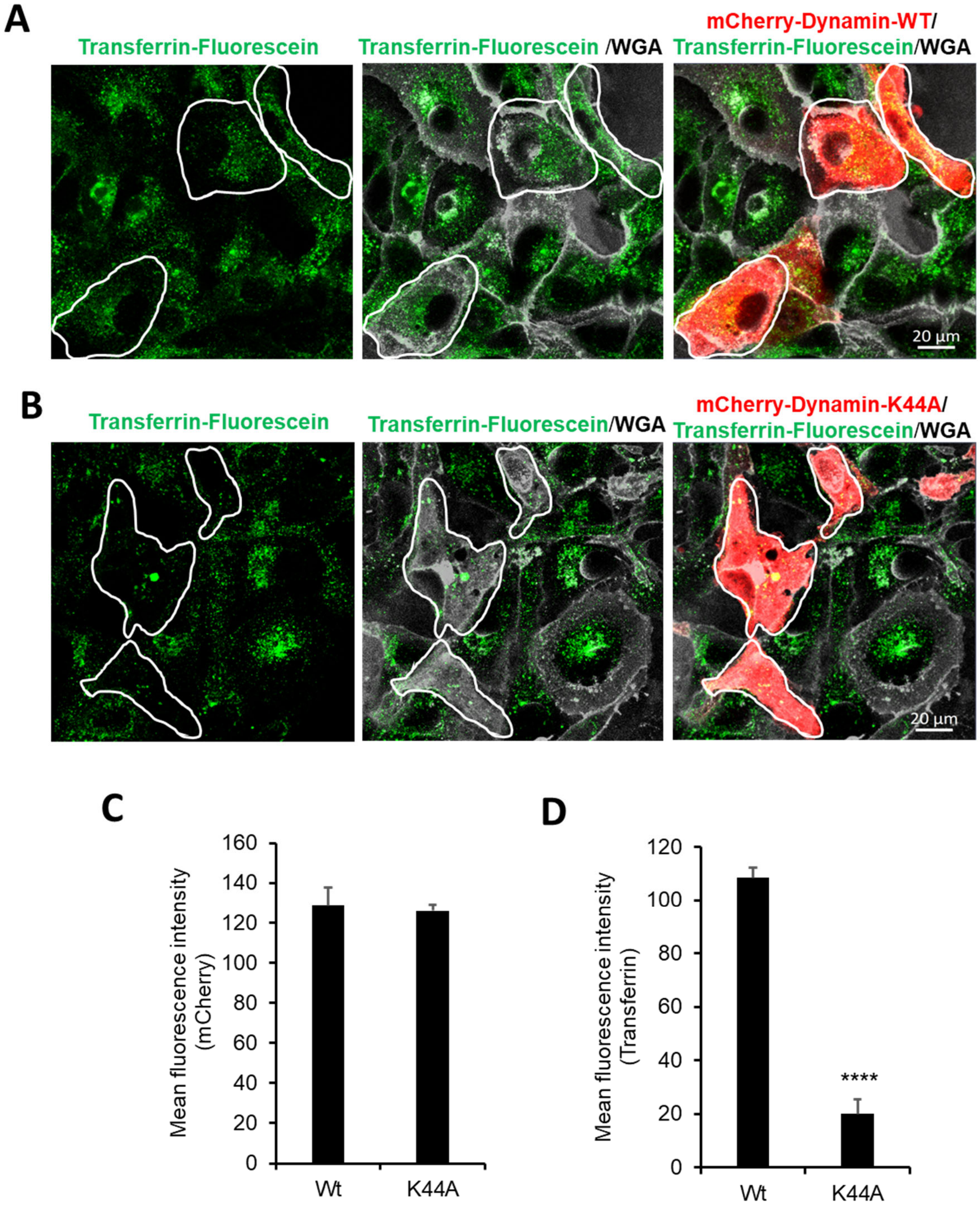

Fig. S7. Validation of Dynamin-2 K44A mutant. Representative confocal images of TZM-bl cells expressing either mCherry-Dynamin-2-WT (A) or mCherry-Dynamin-2-K44A (B), that were incubated with transferrin-fluorescein (as described in Methods) and fixed with 4% PFA. Cell membrane was stained with WGA. (C-D) The mean intensity of mCherry (C) or transferrin-fluorescein (D) was quantified by selecting whole cell area from 8-10 cells (obtained from 3-4 different imaging fields) and plotted in the bar graph.

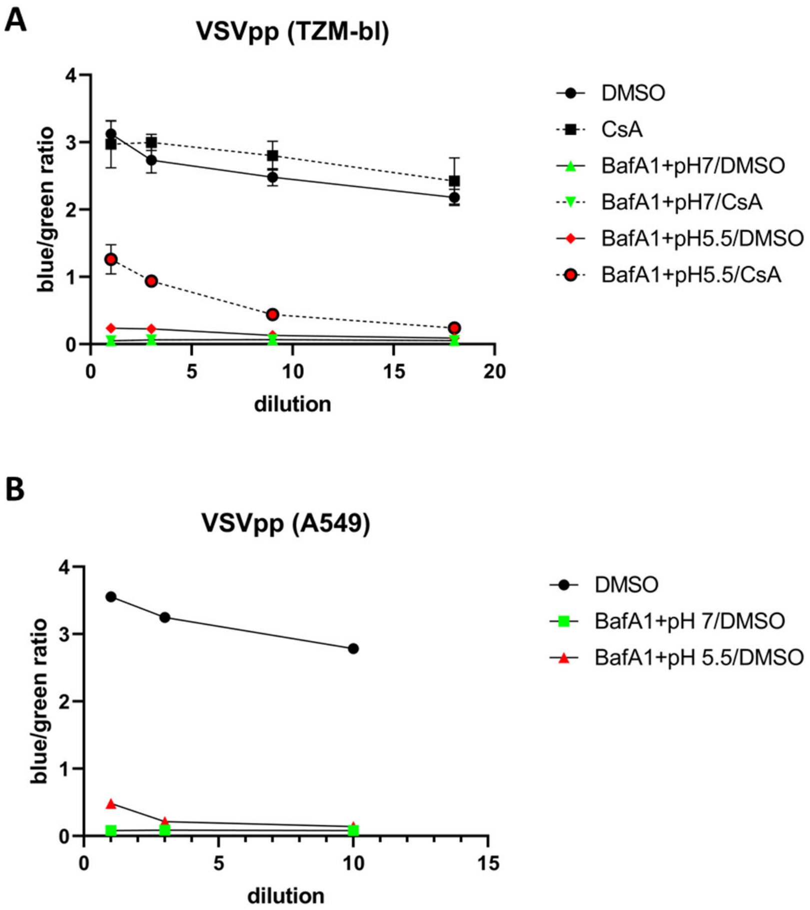

Fig. S8. External pH-induced ("forced") fusion of VSVpp and IAVpp with the cell plasma membrane. TZM-bl (A) and A549 (B) cells were preincubated with 0.1 μM BafA1 for 1 hour to raise endosomal pH before infecting with varied dilutions (as indicated) of HIV-1 particles pseudotyped with VSV-G protein (VSVpp) and containing BlaM-Vpr for virus-cell fusion. TZM-bl cells expressing endogenous levels of IFITMs were preincubated with 20 μM cyclosporine A (CsA) for 1 hour (or with DMSO in control) to enhance viral fusion. VSVpp were pre-bound to TZM-bl or A549 cells in the cold, unbound viruses washed away, and viral fusion with the PM was triggered by applying a prewarmed pH 5.5 citrate buffer. After incubation at 37 ºC for 20 min, acidic buffer was replaced with phenol red-free DMEM/10% FBS, and cells were further incubated for 30 min. (As a control, cells were mock treated with a pH 7.0 buffer). For reference, cells were infected with the same dilutions of pseudoviruses *via* a conventional route by incubating with viruses without BafA1. Following low pH treatment and incubation at neutral pH, cells were loaded with a FRET-based BlaM substrate and incubated overnight at 11 ºC to allow substrate cleavage but block further viral fusion. Ratios of blue and green fluorescence reflective of the cytosolic BlaM-Vpr activity were measured using a plate reader. Note that, due to the low efficiency of forced VSVpp fusion with cells, high virus inputs were used to get above background signals. These conditions lead to BlaM signal saturation for viral fusion through a conventional pathway, as evidenced by a shallow virus dose-dependence. Mean values and SD from triplicate measurements are shown.

Movie Legend

Movie S1: JRFLpp fusion event with pH-neutral vesicles in TZM-bl cell. Single JRFLpp fusion with a pH-neutral endosomal compartment is manifested by a loss of iCherry (red) at 55 min 29 sec min after initiating infection, without losing EcpH (green) or DiD (blue) signals. The frame rate is slowed down around the fusion time to better illustrate the fusion event. Movie is related to Fig. 2A.

Movie S2: JRFLpp fusion with the plasma membrane of TZM-bl cell. Single JRFLpp fusion with the plasma membrane is manifested in a synchronized loss of iCherry (red) and DiD (blue) at 3 min 27 sec min without a loss of EcpH signal. Movie is related to Fig. 2B.

Movie S3: JRFLpp hemifusion with the plasma membrane of TZM-bl cell. Single JRFLpp hemifusion with the plasma membrane is manifested in DiD (blue) loss (white arrow) at 2 min 16 sec without loss of the viral content (iCherry, red) or quenching the EcpH (green) signal. The frame rate is slowed down 2-fold around the DiD loss time (0-6 min). Movie is related to Fig. 2C.

Movie S4: HXB2pp fusion with pH-neutral vesicles in TZM-bl cell. Single HXB2pp fusion with a pH-neutral endosomal compartment is manifested in a loss of iCherry (red, white arrow) at 3 min 26 sec without loss of EcpH (green) or DiD (blue) markers. The frame rate is slowed down to 2-fold (around 0-10 min). Movie is related to Fig. 3A.

Movie S5: HXB2pp fusion with the plasma membrane of TZM-bl cell. Single HXB2pp fusion with the plasma membrane is manifested in a synchronized loss of iCherry (red) and DiD (blue) at 1 min 33 sec, without a loss of EcpH (green) signal. Movie is related to Fig. 3B.

Movie S6: JRFLpp entry into acidified endosome of TZM-bl cell without fusion. Two JRFLpp (marked B' and B") exhibit EcpH quenching (green, arrow) at 15 min 6 sec and 37 min 9 sec, respectively, without changes in the DiD (blue) signal or iCherry (red) signals, demonstrating the particle entry into acidic endosomes. Movie is related to Fig. S2B.

Movie S7: VSVpp fusion in acidified endosome of TZM-bl cell. Single VSVpp entry into acidified endosomes is manifested in EcpH quenching (green, arrow) at 2 min 58 sec and is followed by fusion (loss of iCherry, red) at 5 min16 sec, without a loss of DiD signal (blue). Movie is related to Fig. 5.

Movies S8 and S9: HXB2pp fusion events with pH-neutral vesicles in primary human CD4+ T-cell. In movie 8, single HXB2pp fusion is manifested by iCherry (red) loss at early time point (2 min 91 sec), while movie 9 illustrates a late endosomal fusion event occurring at 79 min 55 sec. In both cases, the EcpH (green) and DiD (red) signals remain unchanged, suggesting the fusion site for HXB2pp in primary human CD4+ T-cells is with pH-neutral vesicles. Movies are related to Fig. 6A and B.

Movie S10: HXB2pp fusion with the plasma membrane of primary human CD4+ T-cell. Single HXB2pp fusion at the cell surface is manifested in a synchronized loss of iCherry (red) and DiD (blue) at 7 min 11 sec, without a loss of EcpH signal (green). Movie is related to Fig. 6C.

Movie S11: JRFLpp fusion with a pH-neutral vesicle in CEM-CCR5 cell. Single JRFLpp fusion with a pH-neutral vesicle is manifested in a loss of iCherry (red) at 18 min 20 sec without changes in the EcpH (green) and DiD (blue) signals. The frame rate is slowed down around the fusion time (11-25 min) to better illustrate this event. Movie is related to Fig. S5A.

Movie S12: JRFLpp fusion with the plasma membrane of CEM-CCR5 cell. Single JRFLpp hemifusion and fusion with the plasma membrane is manifested in a sequential loss of DiD (blue) at 10 min 15 sec and iCherry (red) at 13 min 39 sec, without loss of EcpH fluorescence (green). Movie is related to Fig. S5B.

Movie S13: JRFLpp hemifusion in the plasma membrane of CEM-CCR5 cell. Single JRFLpp hemifusion is manifested in a loss of DiD (blue, arrow) at 3 min 48 sec without loss of iCherry (red) or EcpH (green). Movie is related to Fig. S5C.

Movies S14 and S15: JRFLpp fusion with endosomes in TZM-bl cells expressing iCherry-Dynamin-2 WT. Single JRFLpp (marked white arrow) co-labeled with Gag-iGFP (green) and DiD (blue) fusion with the endosomal membrane is manifested in a loss of a fluid-phase iGFP marker at 2 min 39 sec, without loss of DiD (red) fluorescence. Movie 14 shows only the two viral markers (Gag-iGFP and DiD), and movie 15 shows all three channels, including the iCherry-Dynamin-2 WT (red). Movie is related to Fig. 9A.

Movie S16: JRFLpp hemifusion with TZM-bl cell expressing the dominant-negative dynamin2 mutant, iCherry-Dynamin-2 K44A. Single JRFLpp (marked by white arrow) co-labeled with Gag-iGFP (green) and DiD (blue) hemifuses with the plasma membrane, as evidenced by a loss of DiD (blue) at 26 min 10 sec, without loss of the fluid-phase iGFP marker. iCherry-Dynamin-2 K44A mutant is colored red. Movie is related to Fig. 9B.
